# BAR: Blockwise Adaptive Recoding for Batched Network Coding †

**DOI:** 10.3390/e25071054

**Published:** 2023-07-13

**Authors:** Hoover H. F. Yin, Shenghao Yang, Qiaoqiao Zhou, Lily M. L. Yung, Ka Hei Ng

**Affiliations:** 1 Department of Information Engineering, The Chinese University of Hong Kong, Shatin, New Territories, Hong Kong, China; 2 Department of Electronic and Computer Engineering, The Hong Kong University of Science and Technology, Clear Water Bay, Kowloon, Hong Kong, China; 3 School of Science and Engineering, The Chinese University of Hong Kong, Shenzhen, Shenzhen 518172, China; 4 Department of Computer Science, School of Computing, National University of Singapore, Singapore 119391, Singapore; zhouqq@comp.nus.edu.sg; 5 Independent Researcher, Hong Kong, China; lily@link.cuhk.edu.hk (L.M.L.Y.); kaheicanaan@link.cuhk.edu.hk (K.H.N.)

**Keywords:** linear network coding, batched network coding, adaptive recoding

## Abstract

Multi-hop networks have become popular network topologies in various emerging Internet of Things (IoT) applications. Batched network coding (BNC) is a solution to reliable communications in such networks with packet loss. By grouping packets into small batches and restricting recoding to the packets belonging to the same batch; BNC has much smaller computational and storage requirements at intermediate nodes compared with direct application of random linear network coding. In this paper, we discuss a practical recoding scheme called blockwise adaptive recoding (BAR) which learns the latest channel knowledge from short observations so that BAR can adapt to fluctuations in channel conditions. Due to the low computational power of remote IoT devices, we focus on investigating practical concerns such as how to implement efficient BAR algorithms. We also design and investigate feedback schemes for BAR under imperfect feedback systems. Our numerical evaluations show that BAR has significant throughput gain for small batch sizes compared with existing baseline recoding schemes. More importantly, this gain is insensitive to inaccurate channel knowledge. This encouraging result suggests that BAR is suitable to be used in practice as the exact channel model and its parameters could be unknown and subject to changes from time to time.

## 1. Introduction

Noise, interference and congestion are common causes of packet loss in network communications. Usually, a packet has to travel through multiple hops before it can arrive at the destination node. Traditionally, the intermediate nodes apply the store-and-forward strategy. In order to maintain a reliable communication, retransmission is a common practice. A feedback mechanism is applied so that a network node can acknowledge that a packet is lost. However, due to the delay and bandwidth consumption of the feedback packets, retransmission schemes come with a cost of degraded system performance.

In the era of the Internet of things (IoT), there is a diversity of devices and network topologies. Embedded devices or microcomputers have been heavily deployed due to their mobility, lightweight design, and low power consumption. Multi-hop wireless networks have become a common network topology, highlighting the issues in reliable transmission as wireless links are more vulnerable to packet loss. The packet loss at each link accumulates and the chance of successfully receiving a packet at the destination drops exponentially. Fountain codes, such as in [1,2,3], can recover the lost packets without the need for feedback because of their ratelessness property. However, the throughput still degrades quickly if there is packet loss at each network link unless link-by-link feedback and retransmission are adopted.

### 1.1. Network Coding-Based Approaches

Random linear network coding (RLNC) [4,5], a simple realization of network coding [6,7,8], can achieve the capacity of multi-hop networks with packet loss even without the need for feedback [9,10]. Unfortunately, a direct application of RLNC induces an enormous overhead for the coefficient vectors, as well as high computational and storage costs in network coding operations at intermediate nodes, where the intermediate nodes are usually routers or embedded devices with low computational power and storage space.

Generation-based RLNC was proposed in [11] to resolve these issues. The input packets of the file are partitioned into multiple subsets called the generations, and RLNC is applied to each generation independently. This approach, however, cannot achieve an optimal theoretical rate. Practical concerns and solutions have been further investigated to improve this type of RLNC, such as decoding delay and complexity [12,13,14,15,16,17,18,19,20], packet size [21,22,23,24,25], and coefficient overhead [26,27,28].

Instead of partitioning into disjoint subsets, overlapped subsets were investigated in [29,30,31,32]. To further reduce the computational costs, the use of RLNC was restricted to small subsets of coded packets generated from the input packets in [33,34,35,36,37,38]. Example codes used for generating the coded packets include LDPC and fountain codes. This combination of coding theory and network coding is another variant of RLNC called batched network coding (BNC). BATS codes [38,39], a class of BNC, have a close-to-optimal achievable rate where the achievable rate is upper bounded by the expectation of the rank distribution of the batch transfer matrices that model the end-to-end network operations (packet erasures, network coding operations, etc.) on the batches [40]. This hints that the network coding operations, also known as recoding, have an impact on the throughput of BNC.

### 1.2. Challenges of Recoding in Practice

Baseline recoding is the simplest recoding scheme which generates the same number of recoded packets for every batch. Due to its simple and deterministic structure, baseline recoding appears in many BNC designs and analyses, such as [41,42,43,44,45,46]. However, the throughput of baseline recoding is not optimal with finite batch sizes [47]. The idea of adaptive recoding, aiming to outperform baseline recoding by generating different numbers of recoded packets for different batches, was proposed in [47] without truly optimizing the numbers. Two adaptive recoding optimization models for independent packet loss channels were then formulated independently in [48,49]. A unified adaptive recoding framework was proposed in [50], subsuming both optimization models and supporting other channel models under certain conditions.

Although adaptive recoding can be applied distributively with local network information, it is a challenge to obtain accurate local information when deploying adaptive recoding in real-world scenarios. Adaptive recoding requires two pieces of information: information distribution remaining in the received batches and the channel condition of the outgoing link.

The first piece of information may change over time if the channel condition of the incoming link varies. One reason for the variation is that the link quality can be affected by interference from users of other networks around the network node. We proposed a simple way to adapt to this variation in [49], grouping a few batches into a block and observing the distribution of received batches in this block. This approach was later called blockwise adaptive recoding (BAR) in [51,52].

The second piece of information may also vary from time to time. In some scenarios, such as deep-space [53,54,55] and underwater communications [56,57,58], feedback can be expensive or is not available; therefore, a feedbackless network is preferred. Without feedback, we cannot update our knowledge on the channel condition of the outgoing link. Although we may assume an unchanged channel condition and measure information such as the packet loss rate of the channel beforehand, this measurement, however, can be inaccurate due to observational errors or precision limits.

### 1.3. Contributions

In this paper, we focus on the practical design of applying BAR in real-world applications. Specifically, we answer the following questions in this paper:

1. How does the block size affect the throughput?
2. Is BAR sensitive to an inaccurate channel condition?
3. How can one calculate the components of BAR and solve the optimization efficiently?
4. How can one make use of link-by-link feedback if it is available?

The first question is related to the trade-off between throughput and latency: a larger block induces a longer delay but gives a higher throughput. We show by numerical evaluations that a small block size can already give a significant throughput gain compared with baseline recoding.

For the second question, we demonstrate that BAR performs very well with an independent packet loss model on channels with dependent packet loss. We also show that BAR is insensitive to an inaccurate packet loss rate. This is an encouraging result as this suggests that it is feasible to apply BAR in real-world applications.

The third question is important in practice as BAR is suppose to run at network nodes, usually routers or IoT devices with limited computational power, but also they may need to handle a huge amount of network traffic. Furthermore, by updating the knowledge of the incoming link from a short observation, we need to recalculate the components of BAR and solve the optimization problem again. In light of this, we want to reduce the number of computations to improve the reaction time and reduce the stress of congestion. We answer this question by proposing an on-demand dynamic programming approach to build the components and some implementation techniques to speed up the algorithm for BAR.

Lastly, for the fourth question, we consider both a perfect feedback system (e.g., the feedback passes through a side-channel with no packet loss) and a lossy feedback system (e.g., the feedback uses the reverse direction of the lossy channel for data transmission). We investigate a few ways to estimate the packet loss rate and show that the throughput can be further boosted by using feedback. Furthermore, a rough estimation is sufficient to catch up the variation in the channel condition. In other words, unless there is another application which requires a more accurate estimation on the packet loss rate, we may consider using an estimation with low computational cost, e.g., the maximum likelihood estimator.

### 1.4. Paper Organization and Nomenclature

The paper is organized as follows. We first formulate BAR in Section 2. Then, we discuss the implementation techniques for solving BAR efficiently and evaluate the throughput of different block sizes in Section 3. In Section 4, we demonstrate that BAR is insensitive to inaccurate channel models and investigate the use of feedback mechanisms. Lastly, we conclude the paper in Section 5.

Some specific terminology and notations appear frequently throughout the paper. We summarize some of the important terminology and frequently used notations in Table 1 and Table 2, respectively.

## 2. Blockwise Adaptive Recoding

In this section, we briefly introduce BNC and then formulate BAR.

### 2.1. Network Model

As some intermediate nodes may be hardware-implemented routers or not easily reachable for an upgrade, it is not required to deploy a BNC recoder at every intermediate node. The nodes that do not deploy a recoder are transparent to the BNC network as no network coding operations are performed. In the following text, we only consider intermediate nodes that have deployed BNC recoders.

It is not practical to assume every intermediate node knows the information of the whole network; thus, a distributed scheme that only requires local information is desirable. For example, the statistics of the incoming batches, the channel condition of the outgoing link, etc. In a general network, there may be more than one possible outgoing link to reach the destination. We can assign one recoder or one management unit for each outgoing link at an intermediate node [59,60]. In this way, we need a constraint to limit the number of recoded packets of certain batches sent via the outgoing links. The details are discussed in Section 2.4. In other words, we consider each route from the source to the destination separately as a line network.

**Figure 1:**
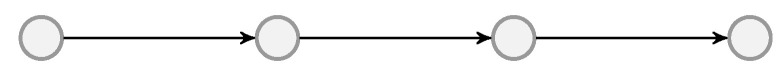
A three-hop line network. Network links only exist between two neighbouring nodes.

Line networks are the fundamental building blocks of a general network. Conversely, a recoding scheme for line networks can be extended to general unicast networks and certain multicast networks [38,48]. A line network is a sequence of network nodes where the network links only exist between two neighbouring nodes. An example of a line network is illustrated in Figure 1. In this paper, we only consider line networks in our numerical evaluations.

### 2.2. Batched Network Coding

Suppose we want to send a file from a source node to a destination node through a multi-hop network. The file is divided into multiple input packets, where each packet is regarded as a vector over a fixed finite field. A BNC has three main components: the encoder, the recoder and the decoder.

The encoder of a BNC is applied at the source node to generate batches from the input packets, where each batch consists of a small number of coded packets. Recently, a reinforcement learning approach to optimize the generation of batches was proposed in [61]. Nevertheless, batches are commonly generated using the traditional approach as follows. To generate a batch, the encoder samples a predefined degree distribution to obtain a degree, where the degree is the number of input packets that constitute the batch. Depending on the application, there are various ways to formulate the degree distribution [62,63,64,65]. According to the degree, a set of packets is chosen randomly from the input packets. The size of the input packets may be obtained via certain optimizations, such as in [66], to minimize the overhead. Each packet in the batch is formed by taking random linear combinations on the chosen set of packets. The encoder generates *M* packets per batch, where *M* is known as the batch size.

Each packet in a batch has a coefficient vector attached to it. Two packets in a batch are defined as linearly independent of each other if and only if their coefficient vectors are linearly independent from each other. Immediately after a batch is generated, the packets within it are assigned as linearly independent from each other. This is accomplished by suitably choosing the initial coefficient vectors [59,67].

A recoder is applied at each intermediate node, performing network coding operations on the received batches to generate recoded packets. This procedure is known as recoding. Some packets of a batch may be lost when they pass through a network link. Each recoded packet of a batch is formed by taking a random linear combination of the received packets in a given batch. The number of recoded packets depends on the recoding scheme. For example, baseline recoding generates the same number of recoded packets for every batch. Optionally, we can also apply a recoder at the source node so that we can have more than *M* packets per batch at the beginning. After recoding, the recoded packets are sent to the next network node.

At the destination node, a decoder is applied to recover the input packets. Depending on the specific BNC, we can use different decoding algorithms, such as Gaussian elimination, belief propagation and inactivation [68,69].

### 2.3. Expected Rank Functions

The rank of a batch at a network node is defined by the number of linearly independent packets remaining in the batch, a measure of the amount of information carried by the batch. Adaptive recoding aims to maximize the sum of the expected value of the rank distribution of each batch arriving at the next network node. For simplicity, we called this expected value the expected rank.

For batch *b*, we denote its rank by $r_{b}$ and the number of recoded packets to be generated by $t_{b}$. The expectation of $r_{b}$ at the next network node, denoted as $E(r_{b},t_{b})$, is known as the expected rank function. We have

(1) $$E\left(r,t\right)=\sum_{i=0}^{t}\mathrm{Pr}\left(X_{t}=i\right)\sum_{j=0}^{\min\{i,r\}}j\zeta_{j}^{i,r},$$

where $X_{t}$ is the random variable of the number of packets of a batch received by the next network node when we send *t* packets for this batch at the current node, and $\zeta_{j}^{i,r}$ is the probability that a batch of rank *r* at the current node with *i* received packets at the next network node has rank *j* at the next network node. The exact formulation of $\zeta_{j}^{i,r}$ can be found in [38], which is $\zeta_{j}^{i,r}=\frac{\zeta_{j}^{i}\zeta_{j}^{r}}{\zeta_{j}^{j}q^{\left(i-j)(r-j\right)}}$, where *q* is the field size for the linear algebra operations and $\zeta_{j}^{m}=\prod_{k=0}^{j-1}\left(1-q^{-m+k}\right)$. It is convenient to use $q=2^{8}$ in practice as each symbol in this field can be represented by 1 byte. For a sufficiently large field size, say $q=2^{8}$, $\zeta_{j}^{i,r}$ is very close to 1 if j=min{i,r}, and is very close to 0 otherwise. That is, we can approximate $\zeta_{j}^{i,r}$ by $\delta_{j,\min\{i,r\}}$ where $\delta_{\cdot ,\cdot }$ is the Kronecker delta. This approximation has also been used in the literature, see, e.g., [45,70,71,72,73,74,75,76].

Besides generating all recoded packets by taking random linear combinations, systematic recoding [39,47,59,67], which concerns received packets as recoded packets, can be applied to save computational time. Systematic recoding can achieve a nearly indistinguishable performance compared with methods which generate all recoded packets by taking random linear combinations [39]. Therefore, we can also use (1) to approximate the expected rank functions for systematic recoding accurately.

For the independent packet loss model with packet loss rate *p*, we have $X_{t}∼\mathrm{Binom}\left(t,1-p\right)$, a binomial distribution. If p=1, then a store-and-forward technique can guarantee the maximal expected rank. If p=0, then no matter how many packets we transmit, the next network node must receive no packets. Thus, we assume 0<p<1 in this paper. It is easy to prove that the results in this paper are also valid for p=0 or 1 when we define $0^{0}:=1$, which is a convention in combinatorics such that Binom(t,0) and Binom(t,1) are well-defined with correct interpretation. In the remaining text, we assume $\zeta_{j}^{i,r}=\delta_{j,\min\{i,r\}}$. That is, for the independent packet loss model, we have

(2) Eindep(r,t)=∑i=0tti(1−p)ipt−imin{i,r}.

A demonstration of the accuracy of the approximation $\zeta_{j}^{i,r}\approx \delta_{j,\min\{i,r\}}$ can be found in Appendix A.

**Figure 2:**
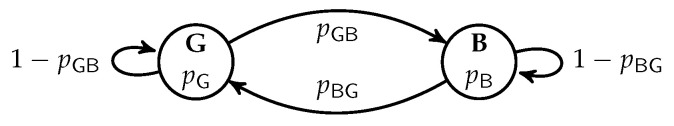
A Gilbert–Elliott (GE) model. In each state, there is an independent event to decide whether a packet is lost or not.

We also consider the expected rank functions for burst packet loss channels modelled by Gilbert–Elliott (GE) models [77,78], where the GE model was also used in other BNC literature such as [52,55,70]. A GE model is a two-state Markov chain, as illustrated in Figure 2. In each state, there is an independent event to decide whether a packet is lost or not. We define $f\left(s,i,t\right):=\mathrm{Pr}\left(S_{t}=s,X_{t}=i\right)$, where $S_{t}$ is the random variable of the state of the GE model after sending *t* packets of a batch. By exploiting the structure of the GE model, computation of *f* can be performed by dynamic programming. Then, we have

(3) $$E_{\mathrm{GE}}\left(r,t\right)=\sum_{i=0}^{t}\left(f\left(G,i,t\right)+f\left(B,i,t\right)\right)\min\left\{i,r\right\}.$$

It is easy to see that it would take more steps to compute (3) than (2). Therefore, a natural question to ask is that for burst packet loss channels, is the throughput gap small between adaptive recoding with (2) and (3)? We demonstrate in Section 4.2 that the gap is small so we use (2) any time a nice throughput is received. Therefore, in our investigation we mainly focus on (2).

In the rest of this paper, we refer to E(r,t) as $E_{\mathrm{indep}}\left(r,t\right)$ unless otherwise specified. From [50], we know that when the loss pattern follows a stationary stochastic process, the expected rank function E(r,t) is a non-negative, monotonically increasing concave function with respect to *t*, which is valid for arbitrary field sizes. Further, E(r,0)=0 for all *r*. However, we need to calculate the values of E(r,t) or its supergradients to apply adaptive recoding in practice. To cope with this issue, we first investigate the recursive formula for E(r,t).

We define the probability mass function of the binomial distribution Binom(t,1−p) by

(4) Bp(t,i)=ti(1−p)ipt−iif0≤i≤t,0otherwise.

For integers r≥0 and t≥−1, we define

(5) βp(t,r)=1ift≤r−1,∑i=0r−1ti(1−p)ipt−i=∑i=0r−1Bp(t,i)otherwise.

When t≥0, the function $\beta_{p}\left(t,r\right)$ is the partial sum of the probability masses of a binomial distribution Binom(t,1−p). The case where t=−1 is used in the approximation scheme in Section 3 and is discussed in that section.

The regularized incomplete beta function, defined as $I_{x}\left(a,b\right):=\frac{\int_{0}^{x}t^{a-1}{\left(1-t\right)}^{b-1}\;dt}{\int_{0}^{1}t^{a-1}{\left(1-t\right)}^{b-1}\;dt}$ ([79], Equation 8.17.2), can be used to express the partial sum of the probability masses of a binomial distribution. When t≥r>0, we can apply ([79], Equation 8.17.5) and obtain

(6) βp(t,r)=∑i=0r−1ti(1−p)ipt−i=Ip(t−r+1,r).

There are different implementations of $I_{p}\left(\cdot ,\cdot \right)$ available for different languages. For example, the GNU Scientific Library [80] for C and C++, or the built-in function betainc in MATLAB. However, most available implementations consider non-negative real parameters and calculate different queries independently. This consideration is too general for our application, as we only need to query the integral points efficiently. In other words, this formula may be sufficient for prototyping or simulation, but it is not efficient enough for real-time deployment on devices with limited computational power. Nevertheless, this formula is useful for proving the following properties:

**Lemma** **1.** *Assume 0<p<1. Let* Λ *be an index set*.

*(a)*  *$B_{p}\left(t+1,i\right)=\left(1-p\right)B_{p}\left(t,i-1\right)+pB_{p}\left(t,i\right)$ for i=0,1,…,t;*
*(b)*  *$\beta_{p}\left(t+1,r\right)\leq \beta_{p}\left(t,r\right)$ where the equality holds if and only if t+1<r or t≥r=0;*
*(c)*  *$\beta_{p}\left(t,r\right)\leq \beta_{p}\left(t+1,r+1\right)$ where the equality holds if and only if t<r;*
*(d)*  *$\beta_{p}\left(t,r+1\right)\geq \beta_{p}\left(t,r\right)$ where the equality holds if and only if t<r;*
*(e)*  *$1\geq \max_{b\in \Lambda }\beta_{p}\left(t_{b},r_{b}\right)\geq \beta_{p}\left(t_{a}+s,r_{a}\right)$ for all a∈Λ and any non-negative integer s;*
*(f)*  *$0\leq \min_{b\in \Lambda }\beta_{p}\left(t_{b},r_{b}\right)\leq \beta_{p}\left(t_{a}-s,r_{a}\right)$ for all a∈Λ and any non-negative integer s such that $t_{a}-s\geq -1$.*

**Proof.**  See Appendix B.    □

With the notation of $\beta_{p}\left(t,r\right)$, we can now write the recursive formula for E(r,t).

**Lemma** **2.** *$E\left(r,t+1\right)=E\left(r,t\right)+\left(1-p\right)\beta_{p}\left(t,r\right)$, where t and r are non-negative integers.*

**Proof.**  Let $Y_{i}$ be independent and identically distributed Bernoulli random variables, where $\mathrm{Pr}(Y_{i}=1)=1-p$ for all *i*. When $Y_{i}=1$, the *i*-th packet is received by the next hop.

When we transmit one more packet at the current node, $Y_{t+1}$ indicates whether this packet is received by the next network node or not. If $Y_{t+1}=0$, i.e., the packet is lost, then the expected rank will not change. If $Y_{t+1}=1$, then the packet is linearly independent from all the already received packets at the next network node if the number of received packets at the next network node is less than *r*. That is, the rank of this batch at the next network node increases by 1 if $\sum_{i=1}^{t}Y_{i}<r$. Therefore, the increment of E(r,t) is $\mathrm{Pr}(Y_{t+1}=1,\sum_{i=1}^{t}Y_{i}<r)$. Note that $\sum_{i=1}^{t}Y_{i}∼\mathrm{Binom}\left(t,1-p\right)$. As $Y_{i}$ are all independent and identically distributed, we have $\mathrm{Pr}\left(Y_{t+1}=1,\sum_{i=1}^{t}Y_{i}<r\right)=\left(1-p\right)\beta_{p}\left(t,r\right)$.    □

The formula shown in Lemma 2 can be interpreted as a newly received packet that is linearly independent of all the already received packets with a probability tends to 1 unless the rank has already reached *r*. This can also be interpreted as $\zeta_{j}^{i,r}=\delta_{j,\min\{i,r\}}$ with a probability tends to 1. The above lemma can be rewritten in a more useful form as stated below.

**Lemma** **3.** *Let t and r be non-negative integers*.

*(a)*  *E(r,t+1)=E(r,t)+(1−p) if t<r;*
*(b)*  *$E\left(r,t\right)=\left(1-p\right)\sum_{j=0}^{t-1}\beta_{p}\left(j,r\right)=\left(1-p\right)\left(\min\left\{r,t\right\}+\sum_{j=r}^{t-1}\beta_{p}\left(j,r\right)\right)$.*

**Proof.**  See Appendix C.    □

### 2.4. Blockwise Adaptive Recoding

The idea of adaptive recoding was presented in [47], and then independently formulated in [48,49]. The former formulation imposes an artificial upper bound on the number of recoded packets and then applies a probabilistic approach to avoid integer programming. The latter investigates the properties of the integer programming problem and proposed efficient algorithms to directly tackle this problem. These two formulations were unified in [50] as a general recoding framework for BNC. This framework requires the distribution of the ranks of the incoming batches, also called the incoming rank distribution. This distribution, however, is not known in advance, and can continually change due to environmental factors. A rank distribution inference approach was proposed in [81], but the long solving time hinders its application in real-time scenarios.

A more direct way to obtain up-to-date statistics is to use the ranks of the few latest batches, a trade-off between a latency of a few batches and the throughput of the whole transmission. This approach was proposed in [49], and later called BAR in [51,52]. In other words, BAR is a recoding scheme which groups batches into blocks and jointly optimizes the number of recoded packets for each batch in the block.

We first describe the adaptive recoding framework and its relation to BAR. We fix an intermediate network node. Let $\left(h_{0},h_{1},\ldots ,h_{M}\right)$ be the incoming rank distribution, $t_{r}$ the number of recoded packets to be sent for a batch of rank *r*, and $t_{\mathrm{avg}}$ the average number of recoded packets to be sent per batch. The value of $t_{r}$ is a non-negative real number that is interpreted as follows. Let $\epsilon =t_{r}-\left\lfloor t_{r}\right\rfloor$ be the fractional part of $t_{r}$. There is an ϵ chance to transmit $\lfloor t_{r}\rfloor +1$ recoded packets, and a 1−ϵ chance to transmit $\left\lfloor t_{r}\right\rfloor$ packets. That is, the fraction is the probability of transmitting one more packet. Similarly, $E(r,t_{r})$ is defined as the linear interpolation by $\left(1-\epsilon \right)E\left(r,\left\lfloor t_{r}\right\rfloor \right)+\epsilon E\left(r,\left\lfloor t_{r}\right\rfloor +1\right)$. The framework maximizes the expected rank of the batches at the next node, which is the optimization problem:

(7) $$\max_{t_{r}\geq 0,\forall r\in \left\{0,1,\ldots ,M\right\}}\sum_{r=0}^{M}h_{r}E\left(r,t_{r}\right)\;s.t.\;\sum_{r=0}^{M}h_{r}t_{r}=t_{\mathrm{avg}}.$$

For BAR, the incoming rank distribution is obtained from the recently received few batches. Let a block be a set of batches. We assume that the blocks at a network node are mutually disjoint. Suppose the node receives a block L. For each batch b∈L, let $r_{b}$ and $t_{b}$ be the rank of *b* and the number of recoded packets to be generated for *b*, respectively. Let $t_{\max}^{L}=t_{\mathrm{avg}}/\left|L\right|$ be the number of recoded packets to be transmitted for the block L. The batches of the same rank are considered individually with the notations $r_{b}$ and $t_{b}$, and the total number of packets to be transmitted for a block is finite; therefore, we assume $t_{b}$ for each b∈L is a non-negative integer, and $t_{\max}^{L}$ is a positive integer. By dividing both the objective and the constraint of the framework by |L|, we obtain the simplest formulation of BAR:

(8) $$\max_{t_{b}\in \left\{0,1,2,\ldots \right\},\forall b\in L}\sum_{b\in L}E\left(r_{b},t_{b}\right)\;s.t.\;\sum_{b\in L}t_{b}=t_{\max}^{L}.$$

To support scenarios with multiple outgoing links for the same batch, e.g., load balancing, we may impose an upper bound on the number of recoded packets per batch. Let $t_{\max}^{b}$ be a non-negative integer that represents the maximum number of recoded packets allowed to be transmitted for the batch *b*. This value may depend on the rank of *b* at the node. Subsequently, we can formulate the following optimization problem based on (8):

(9) $$\begin{array}{ccc} \max_{t_{b}\in \left\{0,1,2,\ldots \right\},\forall b\in L} & \; & \sum_{b\in L}E\left(r_{b},t_{b}\right)\\ s.t. & \; & \sum_{b\in L}t_{b}=t_{\max}^{L}\\ \; & \; & t_{b}\leq t_{\max}^{b},\forall b\in L. \end{array}$$

Note that we must have $\sum_{b\in L}t_{\max}^{b}\geq t_{\max}^{L}$. In the case where this inequality does not hold, we can include more batches in the block to resolve this issue. When $t_{\max}^{b}$ is sufficiently large for all b∈L, (9) degenerates into (8).

The above optimization only depends on the local knowledge at the node. The batch rank $r_{b}$ can be known from the coefficient vectors of the received packets of batch *b*. As a remark, the value of $t_{\max}^{L}$ can affect the stability of the packet buffer. For a general network transmission scenario with multiple transmission sessions, the value of $t_{\max}^{L}$ can be determined by optimizing the utility of certain local network transmissions [82,83].

Though we do not discuss such optimizations in this paper, we consider solving BAR with a general value of $t_{\max}^{L}$.

On the other hand, note that the solution to (9) may not be unique. We only need to obtain one solution for recoding purpose. In general, (9) is a non-linear integer programming problem. A linear programming variant of (9) can be formulated by using a technique in [81]. However, such a formulation has a huge amount of constraints and requires the values of $E(r_{b},t)$ for all b∈L and all possible *t* to be calculated beforehand. We defer the discussion of this formulation to Appendix H.

A network node will keep receiving packets until it has received enough batches to form a block L. A packet buffer is used to store the received packets. Then, the node solves (9) to obtain the number of recoded packets for each batch in the block, i.e., ${\left\{t_{b}\right\}}_{b\in L}$. The node then generates and transmits $t_{b}$-recoded packets for every batch b∈L. At the same time, the network node continually receives new packets. After all the recoded packets for the block L are transmitted, the node drops the block from its packet buffer and then repeats the procedure by considering another block.

We do not specify the transmission order of the packets. Within the same block, the ordering of packets can be shuffled to combat burst loss, e.g., [43,44,52]. Such shuffling can reduce the burst error length experienced by each batch so that the packet loss events are more “independent” from each other. On the other hand, we do not specify the rate control mechanism, as it should be separated as another module in the system. This can be reflected in BAR by choosing suitable expected rank functions, e.g., modifying the parameters in the GE model. BAR is only responsible for deciding the number of recoded packets per batch.

The size of a block depends on its application. For example, if an interleaver is applied to *L* batches, we can group the *L* batches as a block. When |L|=1, the only solution is $t_{b}=t_{\max}^{L}$, which degenerates into baseline recoding. Therefore, we need to use a block size of at least 2 in order to utilize the throughput enhancement of BAR. Intuitively, it is better to optimize (9) with a larger block size. However, the block size is related to the transmission latency as well as the computational and storage burdens at the network nodes. Note that we cannot conclude the exact rank of each batch in a block until the previous network node finishes sending all the packets of this block. That is, we need to wait for the previous network node to send the packets of all the batches in a block until we can solve the optimization problem. Numerical evaluations in Section 3.5 show that |L|=2 already has obvious advantage over |L|=1, and it may not be necessary to use a block size larger than eight.

## 3. Implementation Techniques for Blockwise Adaptive Recoding

In this paper, we focus on the implementation and performance of BAR. Due to the non-linear integer programming structure of (9), we need to make use of certain properties of the model in order to solve it efficiently. The authors of [49] proposed greedy algorithms to solve (9), which were then generalized in [50] to solve (7). The greedy algorithms in [50] have an potential issue when certain probability masses in the incoming rank distribution are too small, as they may take too many iterations to find a feasible solution. The number of iterations is in the order of $\sum_{r=0}^{M}t_{r}$, depending on the solution to (7). That is, we cannot establish a bound on the time complexity as the incoming rank distribution can be arbitrary.

For BAR, we do not have this issue because the number of recoded packets in a block, $t_{\max}^{L}$, is fixed.

In this section, we first discuss the greedy algorithm to solve (9) in Section 3.1. Then, we propose an approximation scheme in Section 3.2, and discuss its application to speed up the solver for practical implementations in Section 3.3. The algorithms in Section 3.1 and Section 3.3 are similar to that in [50], but they are modified to optimize BAR. Note that the algorithms in [50] are generalized from [49], so the correctness of the aforementioned modified algorithms is inherited directly from the generalized proofs in [50]. For the approximation scheme in Section 3.2, which did not appear in [50], a more detailed discussion is provided in this section.

The algorithms in this section frequently query and compare the values of $\left(1-p\right)\beta_{p}\left(t,r\right)$ for different $t\in \{-1,0,1,\ldots ,t_{\max}^{L}\}$ and r∈{0,1,2,…,M}. We suppose a lookup table is constructed so that the queries can be performed in O(1) time. The table is reusable if the packet loss rate of the outgoing link is unchanged. We only consider the subset $\left\{-1,0,1,\ldots ,t_{\max}^{L}\right\}\times \left\{0,1,2,\ldots ,M\right\}$ of the $\beta_{p}$ domain because

1. the maximum rank of a batch is *M*;
2. any $t_{b}$ cannot exceed $t_{\max}^{L}$ as $\sum_{b\in L}t_{b}=t_{\max}^{L}$.

The case t=−1 will be used by our approximation scheme so we keep it in the lookup table. We can build the table on-demand by dynamic programming, discussed in Section 3.4.

### 3.1. Greedy Algorithm

We first discuss the case $t_{\max}^{L}\leq \sum_{b\in L}\min\left\{r_{b},t_{\max}^{b}\right\}$. This condition means that the value of $t_{\max}^{L}$ is too small such that the node has just enough or even not enough time to forward the linearly independent packets received. It is trivial that every ${\left\{t_{b}\right\}}_{b\in L}$ satisfying $0\leq t_{b}\leq \min\left\{r_{b},t_{\max}^{b}\right\}$ and $\sum_{b\in L}t_{b}=t_{\max}^{L}$ is a solution to (9), because every such recoded packet gains 1−p to the expected rank by Lemma 3(a),where this gain is maximal according to the definition of $\beta_{p}\left(t,r\right)$.

For $t_{\max}^{L}>\sum_{b\in L}\min\left\{r_{b},t_{\max}^{b}\right\}$, we can initialize $t_{b}$ by $\min\{r_{b},t_{\max}^{b}\}$ for every b∈L as every such recoded packet gains the maximal value 1−p to the expected rank. After this, the algorithm chooses the batch that can gain the most expected rank by sending one more recoded packet, and assigns one more recoded packet to it. The correctness is due to the concavity of the expected rank functions.

The above initialization reduces most iterations in the algorithm, as in practice, the difference between the number of recoded packets and the rank of the batch is not huge. Algorithm 1 is the improved greedy algorithm. Unlike the version in [50], the complexity of Algorithm 1 does not depend on the solution.

**Algorithm 1:** Solver for BAR.

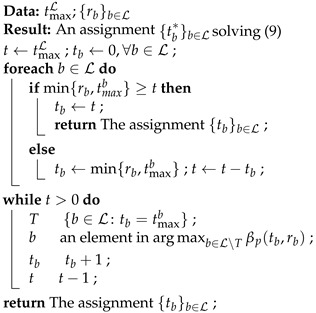

**Theorem** **1.** *Algorithm 1 can be ran in $O(|L|+\max\left\{0,t_{\max}^{L}-\sum_{b\in L}\min\left\{r_{b},t_{\max}^{b}\right\}\right\}\log|L|)$ time.*

**Proof.**  There are totally $\max\{0,t_{\max}^{L}-\sum_{b\in L}\min\left\{r_{b},t_{\max}^{b}\right\}\}$ iterations in the **while** loop. The query of $\mu ={arg max}_{b\in L\setminus T}\beta_{p}\left(t_{b},r_{b}\right)$ can be implemented by using a binary heap. The initialization of the heap, i.e., heapify, takes O(|L|) time, which can be performed outside the loop. Each query in the loop takes O(1) time. The update from $\beta_{p}\left(t_{\mu },r_{\mu }\right)$ into $\beta_{p}\left(t_{\mu }+1,r_{\mu }\right)$, if $t_{\mu }<t_{\max}^{\mu }-1$, takes O(log|L|) time. For $t_{\mu }=t_{\max}^{\mu }-1$, we may remove the entry from the heap, taking the same time complexity as the update above. As $\sum_{b\in L}\min\left\{r_{b},t_{\max}^{b}\right\}\geq t_{\max}^{L}$ by assumption, the algorithm will not query an empty heap. Therefore, the overall time complexity is $O(|L|+\max\left\{0,t_{\max}^{L}-\sum_{b\in L}\min\left\{r_{b},t_{\max}^{b}\right\}\right\}\log|L|)$.    □

In the algorithm, we assume that a lookup table for $\beta_{p}\left(t,r\right)$ is pre-computed. The table can be reused unless there is an update on the outgoing channel condition. Nevertheless, we will discuss an efficient way to construct the lookup table in Section 3.4, and the insignificance of the measurement or prediction errors of the loss probability of the outgoing channel in Section 4.

As the query ${arg max}_{b\in L\setminus T}\beta_{p}\left(t_{b},r_{b}\right)$ is run repeatedly and an update is performed after every query, we can use a binary heap as described in the proof in real implementation. Note that by Lemma 1(b), $\beta_{p}\left(t_{\mu },r_{\mu }\right)\geq \beta_{p}\left(t_{\mu }+1,r_{\mu }\right)$, so the update is a decrease key operation in a max-heap. In other words, a Fibonacci heap [84] cannot benefit from this operation here.

### 3.2. Equal Opportunity Approximation Scheme

**Figure 3:**
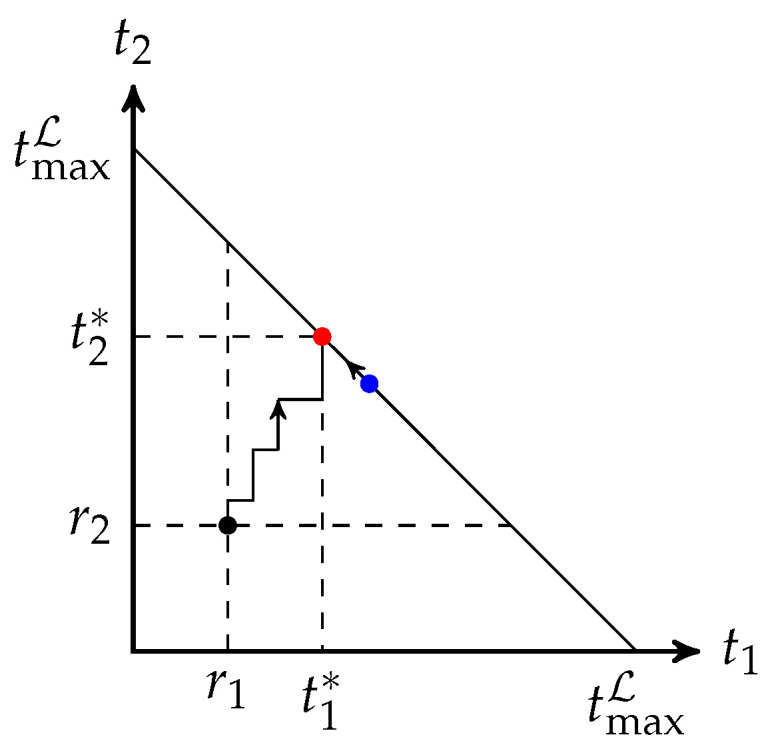
This figure illustrates the idea of modifying the output of an approximation scheme with two batches, where L={1,2}, $t_{\max}^{L}\geq r_{1}+r_{2}$ and $t_{\max}^{1},t_{\max}^{2}\geq t_{\max}^{L}$. The red and blue dots represent the optimal and approximate solutions on the facet $t_{1}+t_{2}=t_{\max}^{L}$, respectively. Algorithm 1 starts the search from an interior point $\left(r_{1},r_{2}\right)$, while a modification approach starts the search from the blue dot.

Algorithm 1 increases $t_{b}$ step by step. From a geometric perspective, the algorithm finds a path from the interior of a compact convex polytope that models the feasible solutions to the facet $H:\sum_{b\in L}t_{b}=t_{\max}^{L}$. If we have a method to move a non-optimal feasible point on H towards an optimal point, together with a fast and accurate approximation to (8) or (9), then we can combine them to solve (9) faster than using Algorithm 1 directly. This idea is illustrated in Figure 3. A generalized tuning scheme can be found in [50] based on the algorithm in [49]. However, there is no approximation scheme proposed in [50].

We first give an approximation scheme in this subsection. The approximation is based on an observation of the solution for (8) that does not impose an upper boundary on $t_{b}$: A batch of higher rank should have more recoded packets transmitted than a batch of lower rank. Unless most $t_{\max}^{b}$ are too small, the approximation for (8) is also a good approximation for (9).

**Theorem** **2.** *Let L be a block where |L|≥2. If ${\left\{t_{b}\right\}}_{b\in L}$ solves* (8) *and $t_{b}\geq r_{b}$ for all b∈L, then $t_{m}<t_{n}$ for all m,n∈L such that $r_{m}<r_{n}$.*

**Proof.**  See Appendix D.    □

As we cannot generate any linearly independent packets for a batch of rank 0, we have E(0,·)=0. Therefore, we can exclude batches of rank 0 from L before we start the approximation. We define $L=\{b\in L:r_{b}>0\}\subseteq L$. When $t_{\max}^{L}>\sum_{b\in L}r_{b}$, we have $t_{b}\geq r_{b}$ for all b∈L. An easy way to obtain an approximation is to assign ${\left\{t_{b}\right\}}_{b\in L}$ following the guidelines given in Theorem 2 by:

- $t_{b}=0$ for all b∈L\L;
- $t_{b}=r_{b}+\ell$ for all b∈L.

where $\ell =(t_{\max}^{L}-\sum_{b\in L}r_{b})/|L|$. In the case where *ℓ* is not an integer, we can round it up for batches with higher ranks and round it down for those with lower ranks.

The above rules allocate the unassigned packets to batches equally after $r_{b}$ packets have been assigned to each batch *b*. Thus, we call this approach the equal opportunity approximation scheme. The steps of this scheme are summarized in Algorithm 2.

Note that we do not need to know the packet loss rate *p* to apply this approximation. That is, if we do not know the value of *p*, we can still apply this approximation to outperform baseline recoding.

**Theorem** **3.** *Algorithm 2 approximates* (8) *in O(|L|) time. If $t_{\max}^{L}\leq \sum_{b\in L}r_{b}$, then the algorithm solves* (8).

**Algorithm 2:** Equal opportunity approximation scheme.

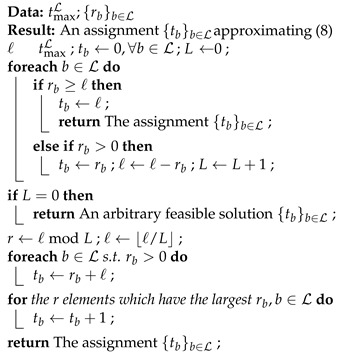

**Proof.**  It is easy to see that Algorithm 2 outputs ${\left\{t_{b}\right\}}_{b\in L}$ which satisfies $\sum_{b\in L}t_{b}=t_{\max}^{L}$. That is, the output is a feasible solution of (8). Note that |L|≤|L|, so the assignments and the branches before the last **for** loop take O(|L|) time in total. The variable *L* after the first **foreach** loop equals |L|. Adding one to the number of recoded packets for r=ℓmodL batches with the highest ranks can be performed in O(|L|) time. Therefore, the overall running time is O(|L|).

If L=∅, i.e., the whole block is lost, then any feasible ${\left\{t_{b}\right\}}_{b\in L}$ is a solution, and the optimal objective value is 0. If $t_{\max}^{L}\leq \sum_{b\in L}r_{b}$, then the algorithm terminates with an output satisfying $t_{b}\leq r_{b}$ for all b∈L, which is an optimal solution.    □

For the step that adds 1 to the number of recoded packets for r=ℓmodL batches with the highest ranks in the algorithm, the worst linear time case can be achieved by using introselect [85] (which is quickselect [86] with a random pivot, but changes to use the median of medians [87] pivot strategy when the complexity grows). We use the selection algorithm to find the *r*-th largest element, making use of its intermediate steps. During an iteration, one of the following three cases will occur. If the algorithm decides to search a part larger than the pivot, then the discarded part does not contain the largest *r* elements. If a part smaller than the pivot is selected, then the discarded part is part of the largest *r* elements. If the pivot is exactly the *r*-th largest element, then the part larger than the pivot together with the pivot are part of the largest *r* elements.

In practice, the batch size *M* is small. We can search these *r* batches with the highest ranks in O(|L|+M) time using a counting technique as an efficient alternative. The technique is to use part of the counting algorithm [88]. We first compute a histogram of the number of times each rank occurs, taking O(M) time for initialization and O(|L|) time to scan the block. Then, we can scan and count the frequencies of the histogram from the highest rank, and eliminate the part where the count exceeds ℓmod|L|. This takes O(M) time. Lastly, we scan the ranks of the batches again in O(|L|) time. If included in the modified histogram, we add 1 to the corresponding $t_{b}$ and minus 1 to the corresponding frequency in the histogram.

Algorithm 2 is a (1−p)-approximation algorithm, although the relative performance guarantee factor 1−p is not tight in general. However, this suggests that the smaller the packet loss rate *p*, the more accurate the output the algorithm gives. We defer this discussion to Appendix E.

### 3.3. Speed-Up via Approximation

In this subsection, we discuss the implementation that corrects an approximate solution to an optimal solution for (9). Algorithm 3 is a greedy algorithm that uses any feasible solution of (8) as a starting point. The **foreach** loop removes the exceeding recoded packets, assigning the released slot to another batch following the iterations in Algorithm 1. This can be regarded as mimicking a replacement of $\beta_{p}\left(\cdot ,\cdot \right)$ with the smallest possible value for the batch *b* that violates the constraint $t_{b}\leq t_{\max}^{b}$. After this, the intermediate solution is a feasible solution to (9). Then, the last loop finds an increase to the objective by reassigning some slots among the batches.

**Algorithm 3:** Solver for BAR via approximation.

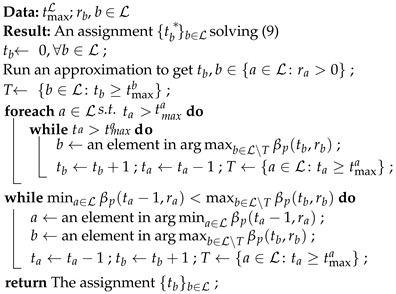



Note that the algorithm may query $\beta_{p}\left(t_{a}-1,r_{a}\right)$ for a∈L. If $t_{a}=0$, then it has access to the value $\beta_{p}\left(-1,r_{a}\right)$. Recall that we defined $\beta_{p}\left(-1,\cdot \right)=1$, the upper bound of $\beta_{p}\left(\cdot ,\cdot \right)$ by (10). Therefore, these values act as barriers to prevent outputting a negative number of recoded packets.

**Theorem** **4.** *Let ${\left\{t_{b}\right\}}_{b\in L}$ be an approximate solution of* (8) computed in $O(T_{\mathrm{approx}})$ time. Algorithm 3 can be run in $O(T_{\mathrm{approx}}+|L|+\sum_{b\in L}|t_{b}^{*}-t_{b}|\log|L|)$ time.

**Proof.**  The assignments before the **foreach** loop takes $O(T_{\mathrm{approx}}+|L|)$ time. There are a total of $\sum_{b\in L}\left|t_{b}^{*}-t_{b}\right|/2$ iterations in the loops. The queries for the minimum and maximum values can be implemented using a min-heap and a max-heap, respectively. Similar to Algorithm 1, we can use binary heaps, taking O(|L|) initialization time, O(1) query time, and O(log|L|) update time. Each iteration contains at most two heap queries and four heap updates. The update of the set *T* can be performed implicitly by setting $\beta_{p}\left(t_{a},r_{a}\right)$ to 0 during the heap updates for a∈T. The overall time complexity is then $O(T_{\mathrm{approx}}+|L|+\sum_{b\in L}|t_{b}^{*}-t_{b}|\log|L|)$. □

In the last **while** loop, we need to query the minimum of $\beta_{p}\left(t_{a}-1,r_{a}\right)$ and the maximum of $\beta_{p}\left(t_{b},r_{b}\right)$. It is clear that we need to decrease the key $\beta_{p}\left(t_{b},r_{b}\right)$ to $\beta_{p}\left(t_{b}+1,r_{b}\right)$ in the max-heap, and increase the key $\beta_{p}\left(t_{a}-1,r_{a}\right)$ to $\beta_{p}\left(t_{a}-2,r_{a}\right)$ in the min-heap. However, we can omit the updates for batches *a* and *b* in the max-heap and min-heap, respectively, i.e., reduce from four heap updates to two heap updates. We defer this discussion to Appendix G.

### 3.4. Construction of the Lookup Table

In the above algorithms, we assume that we have a lookup table for the function $\beta_{p}\left(\cdot ,\cdot \right)$ so that we can query its values quickly. In this subsection, we propose an on-demand approach to construct a lookup table by dynamic programming.

Due to the fact that $\sum_{i=0}^{t}B_{p}\left(t,i\right)=1$, we have

(10) $$0\leq \beta_{p}\left(t,r\right)\leq 1.$$

Furthermore, it is easy to see that

(11) $$\begin{array}{ccccccc} \beta_{p}\left(t,r\right) & = & 0 & \;\mathrm{if}\;\mathrm{and}\;\mathrm{only}\;\mathrm{if} & r & = & 0\;\mathrm{and}\;t\geq 0; \end{array}$$

(12) $$\begin{array}{ccccccc} \beta_{p}\left(t,r\right) & = & 1 & \;\mathrm{if}\;\mathrm{and}\;\mathrm{only}\;\mathrm{if} & t & \leq & r-1. \end{array}$$

**Figure 4:**
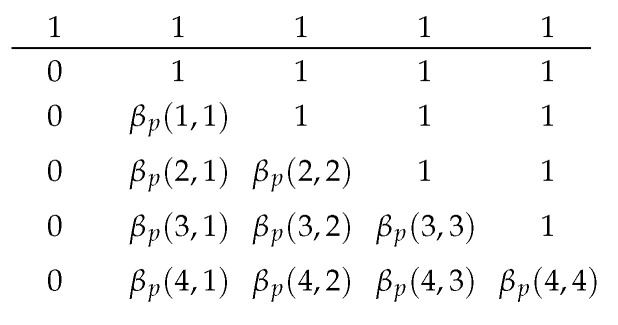
The tabular appearance of the function $\beta_{p}\left(t,r\right)$ after introducing boundaries 0 and 1 s. The rows and columns correspond to t=−1,0,1,… and r=0,1,2,…, respectively. The row above the line is $\beta_{p}\left(-1,\cdot \right)$.

A tabular form of $\beta_{p}$ is illustrated in Figure 4 after introducing the boundaries 0 and 1 s.

**Figure 5:**
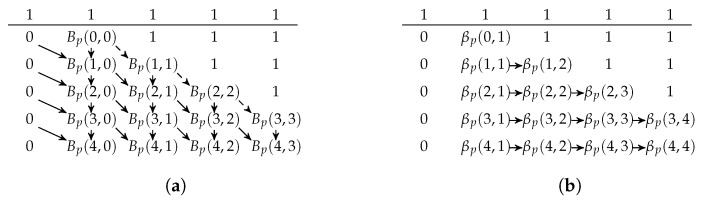
The figures illustrate the two stages of the table generation. The indices start from (−1,0). The first row has the index y=−1, which is the row above the line. Compared to Figure 4, $\beta_{p}\left(0,1\right)=\beta_{p}\left(1,2\right)=\ldots =1$ can be substituted in directly without using the relation (15). (**a**) The first stage of the table generation. The 1 and 0 s paddings are generated first. The solid and dashed arrows represent (13) and (14), respectively. (**b**) The second stage of the table generation. The 1 and 0 s paddings are kept. The arrows represent the recursive relation (15) with the $B_{p}$ function in-place.

Being a dynamic programming approach, we need the following recursive relations:

(13) $$\begin{array}{cccc} B_{p}\left(t+1,r\right) & = & \left(1-p\right)B_{p}\left(t,r-1\right)+pB_{p}\left(t,r\right) & \;\mathrm{for}\;0\leq r\leq t; \end{array}$$

(14) $$\begin{array}{cccc} B_{p}\left(r,r\right) & = & \left(1-p\right)B_{p}\left(r-1,r-1\right) & \;\mathrm{for}\;r>0; \end{array}$$

(15) $$\begin{array}{cccc} \beta_{p}\left(t,r\right) & = & \beta_{p}\left(t,r-1\right)+B_{p}\left(t,r-1\right) & \;\mathrm{for}\;1<r\leq t+1, \end{array}$$

where (13) is stated in Lemma 1(a); and (14) and (15) are by the definitions of $B_{p}\left(t,r\right)$ and $\beta_{p}\left(t,r\right)$, respectively. The boundary conditions are $B_{p}\left(0,0\right)=1$, $B_{p}\left(i,-1\right)=0$, and $\beta_{p}\left(i,1\right)=B_{p}\left(i,0\right)$ for i=0,1,…. The table can be built in-place in two stages. The first stage fills in $B_{p}\left(y,x-1\right)$ at the (y,x) position of the table. The second stage finishes the table by using (15). Figure 5 illustrates the two stages where the arrows represent the recursive relations (13)–(15). As $\beta_{p}\left(0,1\right)=\beta_{p}\left(1,2\right)=\ldots =1$, the corresponding entries can be substituted in directly.

**Figure 6:**
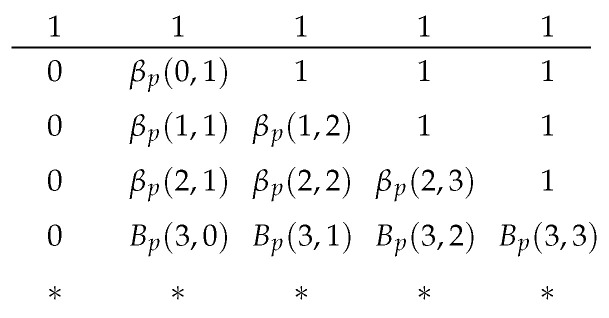
The values prepared when $t_{b^{\prime }}=2$. The asterisks represent the values that are not yet initialized.

We can compute the values in the table on-demand. Suppose we have ${\left\{t_{b}\right\}}_{b\in L}$ in an iteration of Algorithm 1 so that we need the values of $\beta_{p}\left(t_{b},r_{b}\right)$ for b∈L. Let $b^{\prime }$ be an element in ${arg max}_{b\in L}r_{b}$. The table has $r_{b^{\prime }}+1$ columns. By the criteria of selecting *b* in Algorithm 1 and by Lemmas 1(c) and (d),we have $\max_{b\in L}t_{b}=t_{b^{\prime }}$. From Figure 5, we know we have to calculate all rows of β(t,r) for $t\leq t_{b^{\prime }}$. Furthermore, the recursive relations on a row only depend on the previous row; thus, we need to prepare the values of $B_{p}$ in the next row so that we have the values to compute $\beta_{p}$ in the next row. As an example, Figure 6 illustrates the values we have prepared when $t_{b^{\prime }}=2$.

Each entry in the table is modified at most twice during the two stages. Each assignment takes O(1) time. Therefore, the time and space complexities for building the table are both O(MR), where *R* is the number of rows we want to construct. When restricted by the block size, we know that $R\leq t_{\max}^{L}$. The worst case is that we only receive one rank-*M* batch for the whole block, which is unlikely to occur. In this case, we have the worst case complexity $O(Mt_{\max}^{L})$.

Note that we can use fixed-point numbers instead of floating point numbers for a more efficient table construction. Furthermore, the numerical values in the table are not important as long as the orders for any pair of values in the table are the same.

### 3.5. Throughput Evaluations

We now evaluate the performance of BAR in a feedbackless multi-hop network. Note that baseline recoding is a special case of BAR with block size 1. Our main goal here is to show the throughput gain of BAR among different block sizes. In the evaluation, all (recoded) packets of a batch are sent before sending those of another batch.

Let $\left(h_{0},h_{1},\ldots ,h_{M}\right)$ be the incoming rank distribution of batches arriving at a network node. The normalized throughput at a network node is defined as the average rank of the received batches divided by the batch size, i.e., $\sum_{i=0}^{M}ih_{i}/M$. In our evaluations in this subsection, we set $t_{\max}^{L}=M\left|L\right|$ for every block L. That is, the source node transmits *M* packets per batch. We assume that every link in the line network has independent packet loss with the same packet loss rate *p*. In this topology, we set a sufficiently large $t_{\max}^{b}$ for every batch, say, $t_{\max}^{b}=t_{\max}^{L}$.

**Figure 7:**
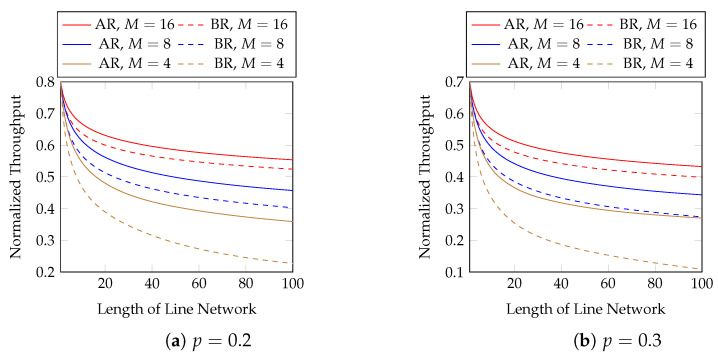
Adaptive recoding (AR) vs. baseline recoding (BR) in line networks of different lengths, batch sizes and packet loss rates.

We first evaluate the normalized throughput with different batch sizes and packet loss rates. Figure 7 compares adaptive recoding (AR) and baseline recoding (BR) when we know the rank distribution of the batches arriving at each network node before the node applies BAR. In other words, Figure 7 shows the best possible throughput of AR. We compare the effect of block sizes later. We observe that

1. AR has a higher throughput than BR under the same setting;
2. the difference in throughput between AR and BR is larger when the batch size is smaller, the packet loss probability is larger, or the length of the line network is longer.

In terms of throughput, the percentage gains of AR over BR using M=4 and p=0.2 are 23.3 and 33.7% at the 20-th and 40-th hops, respectively. They become 43.8 and 70.3%, respectively, when p=0.3.

**Figure 8:**
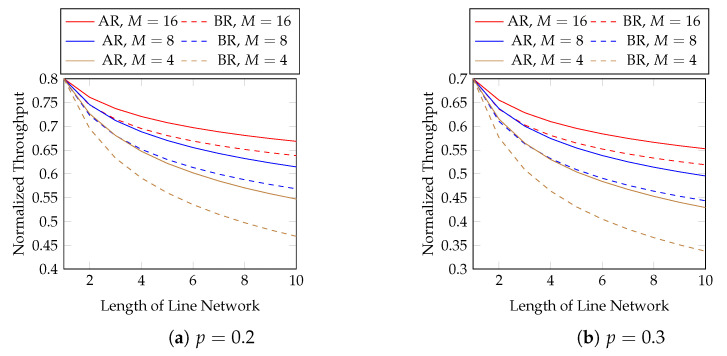
The first 10 hops in Figure 7.

Although the above figure shows that the throughput of BNC with AR maintains a good performance when the length of the line network is long, many applications use a much shorter line network. We zoom into the figure for the first 10 hops in Figure 8 for practical purposes.

**Figure 9:**
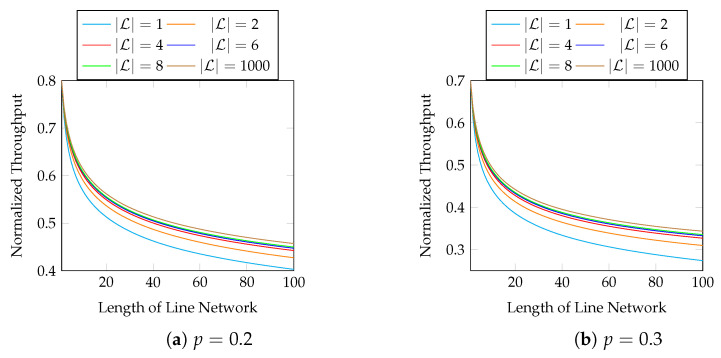
The effect of different block sizes with M=8.

**Figure 10:**
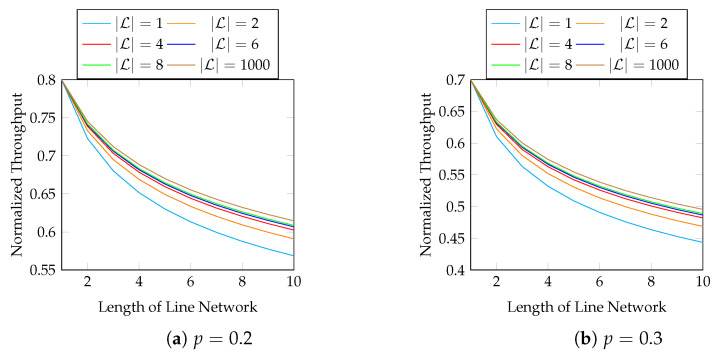
The first 10 hops in Figure 9.

Now, we consider the effect of different block sizes. Figure 9 shows the normalized throughput of different |L| and *p* with M=8. The first 10 hops in Figure 9 are zoomed in in Figure 10. We observe that

1. a larger |L| results a better throughput;
2. using |L|=2 already gives a much larger throughput than using |L|=1;
3. using |L|>8 gives little extra gain in terms of throughput.

**Figure 11:**
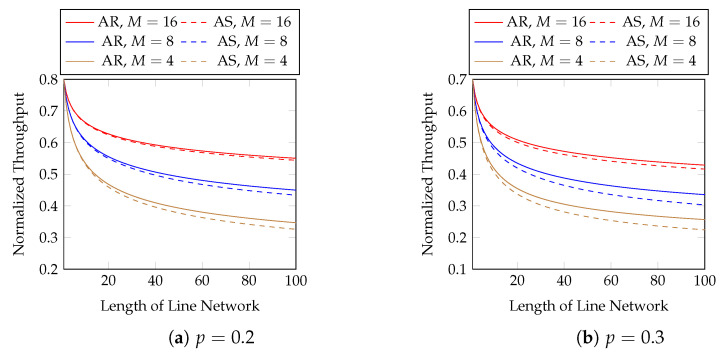
Approximation vs. optimal AR in line networks of different lengths, batch sizes and packet loss rates.

**Figure 12:**
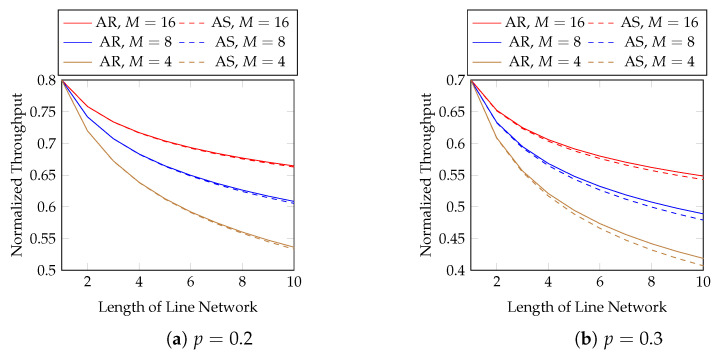
The first 10 hops in Figure 11.

Next, we show the performance of the equal opportunity approximation scheme. Figure 11 compares the normalized throughput achieved by Algorithm 2 (AS) and the true optimal throughput (AR). We compare the best possible throughput of AR here, i.e., the same setting as in Figure 7. The first 10 hops in Figure 11 are zoomed in in Figure 12. We observe that

1. the approximation is close to the optimal solution;
2. the gap in the normalized throughput is smaller when the batch size is larger, the packet loss probability is smaller, or the length of the line network is shorter.

## 4. Impact of Inaccurate Channel Models

In this section, we first demonstrate that the throughput of BAR is insensitive to inaccurate channel models and packet loss rates. Then, we investigate the feedback design and show that although feedback can enhance the throughput, the benefit is insignificant. In other words, BAR works very well without the need of feedback.

### 4.1. Sensitivity of $\beta_{p}\left(t,r\right)$

**Figure 13:**
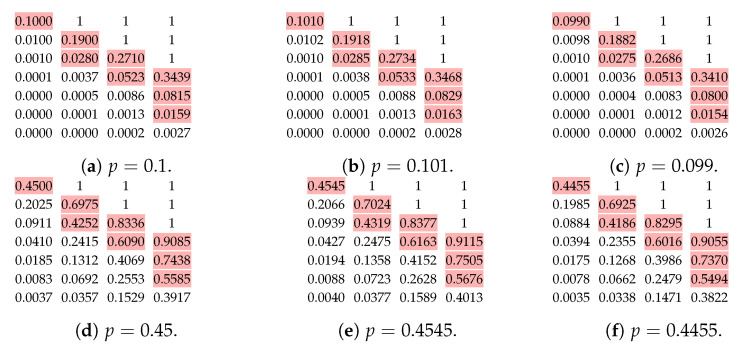
The values of $\beta_{p}\left(t,r\right)$ for r=1,2,3,4 and t=1,2,… with different *p*. The coloured numbers are the largest eight values smaller than 1.

We can see that our algorithms only depend on the order of the values of $\beta_{p}\left(\cdot ,\cdot \right)$; therefore, it is possible that the optimal ${\left\{t_{b}\right\}}_{b\in L}$ for an incorrect *p* is the same for a correct *p*. As shown in Figure 4, the boundaries 0 and 1 s are unaffected by p∈(0,1). That is, we only need to investigate the stability of $\beta_{p}\left(t,r\right)$ for t≥r>0. We calculate values of $\beta_{p}\left(t,r\right)$ corrected to four digital places in Figure 13 for M=4, and p=0.1,0.45 and their 1% relative changes. We can see that the order of the values are mostly the same when we slightly change *p*.

**Figure 14:**
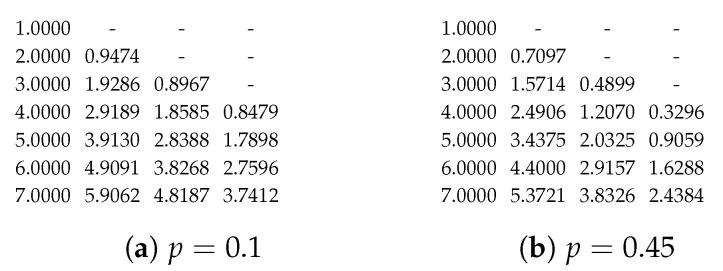
The condition numbers of $\beta_{p}\left(t,r\right)$ for r=1,2,3,4 and t=1,2,….

We can also check with the condition number [89] to verify the stability. Roughly speaking, the relative change in the function output is approximately equal to the condition number times the relative change in the function input. A small condition number of $\beta_{p}\left(t,r\right)$ means that the effect of the inaccurate *p* is small. As shown in Figure 13, the values of $\beta_{p}\left(t,r\right)$ drop quickly when *t* increases. In the view of the throughput, which is proportional to the sum of these values, we can tolerate a larger relative change, i.e., a larger condition number, when $\beta_{p}\left(t,r\right)$ is small. We calculate condition numbers of $\beta_{p}\left(t,r\right)$ in Figure 14 by the formula stated in Theorem 5.

**Theorem** **5.** *Let p∈(0,1) and t≥r>0. The condition number of $\beta_{p}\left(t,r\right)$ with respect to p is $\frac{p^{t-r+1}{\left(1-p\right)}^{r-1}t!}{I_{p}\left(t-r+1,r\right)\left(t-r\right)!\left(r-1\right)!}$, or equivalently, ∑j=0r−1(−1)jr−1jpt−r+j+1∑j=0r−1(−1)jr−1jpt−r+j+1/(t−r+j+1).*

**Proof.**  See Appendix F. □

### 4.2. Impact of Inaccurate Channel Models

To demonstrate the impact of an inaccurate channel model, we consider three different channels to present our observations.

- ch1: independent packet loss with constant loss rate p=0.45.
- ch2: burst packet loss modelled by the GE model illustrated in Figure 2 with the parameters used in [70], namely $p_{GB}=p_{BG}=p_{G}=0.1$, $p_{B}=0.8$.
- ch3: independent packet loss with varying loss rates p=0.45+0.3sin(2πc/1280), where *c* is the number of transmitted batches.

All the three channels have the same average packet loss rate of 0.45. The formula of ch3 is for demonstration purpose only.

We now demonstrate the impact of inaccurate *p* on the throughput. We consider a line network where all the links use the same channel (ch1, ch2, or ch3). In this topology, we set a sufficiently large $t_{\max}^{b}$ for every batch, say, $t_{\max}^{b}=t_{\max}^{L}$. Similar to the previous evaluation, all (recoded) packets of a batch are sent before sending those of another batch. Furthermore, we set $t_{\max}^{L}=M\left|L\right|$ for every block L.

**Figure 15:**
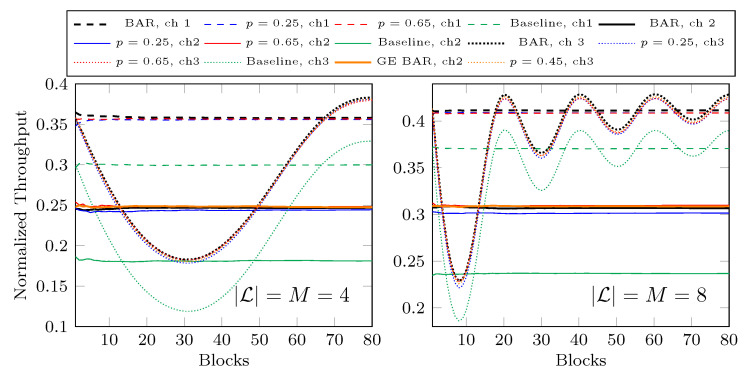
Throughput with inaccurate channel conditions.

In Figure 15 we plot the normalized throughput of the first 80 received blocks at the fourth hop where |L|=M=4 or 8. We use BAR with (2) for each network although ch2 is a bursty channel. The black curves with BAR are the throughput of BAR where the loss rate is known. For ch1 and ch2, this loss rate *p* is a constant of 0.45. The red and blue curves are the throughput of BAR when we guess p=0.65 and 0.25, respectively, which is ±0.2 from the average loss rate of 0.45. As there is no feedback, we do not change our guess on *p* for these curves. We can see that the throughput is actually very close to the corresponding black curves. This suggests that in the view of the throughput, BAR is not sensitive to *p*. Even with a wild guess on *p*, BAR still outperforms BR, as illustrated by the green curves. Regarding ch2, we also plot the orange curve with GE BAR, which is the throughput achieved by BAR with (3). We can see that the gap between the throughput achieved by BAR with (2) and (3) is very small. As a summary of our demonstration:

1. We can use BAR with (2) for bursty channels and the loss in throughput is insignificant.
2. BAR with an inaccurate constant *p* can achieve a throughput close to the one when we have the exact real-time loss rate.
3. We can see a significant throughput gain from BR by using BAR even with inaccurate channel models.

### 4.3. Feedback Design

Although an inaccurate *p* can give an acceptable throughput, we can further enhance the throughput by adapting the varying *p* values. To achieve this goal, we need to use feedback.

We adopt a simple feedback strategy which lets the next node return the number of received packets of the batches for the current node to estimate *p*. Although the next node does not know the number of lost packets per batch, it knows the number of received packets per batch. Therefore, we do not need to introduce more overhead to the transmitted packets by the current node.

When we estimate *p*, we have to know the number of packets lost during a certain time frame. If the time frame is too small, the estimation is too sensitive so the estimated *p* changes rapidly and unpredictably. If the time frame is too long, we captured too much out-dated information about the channel so the estimated *p* changes too slowly and may not be able to adapt to the real loss rate. Recall that the block size is not large as we want to keep the delay small. We use a block as an atomic unit of the time frame. The next node gives feedback on the number of received packets per block. The current node uses the feedback of the blocks in the time frame to estimate *p*. We perform an estimation of *p* per received feedback. In this way, the estimated *p* is the same for each block so that we can apply BAR with (2).

If the feedback is sent via a reliable side channel, then we can assume that the current node can always receive the feedback. However, if the feedback is sent via an unreliable channel, say, the reverse direction of the same channel the data packets were sent from, then we need to consider feedback loss. Let Λ be a set of blocks in a time frame with received feedback. We handle the case of feedback loss by considering the total number of packets transmitted for the blocks in Λ as the total number of packets transmitted during the time frame. In this way, we can also start the estimation before a node sends enough blocks to fill up a time frame. Suppose no feedback is received for every block in a time frame, then we reuse the previously estimated *p* for BAR.

At the beginning of the transmission, we have no feedback yet so we have no information to estimate *p*. To outperform BR without the knowledge of *p*, we can use the approximation of BAR given by Algorithm 2. Once we have received at least one feedback, we can then start estimating *p*.

### 4.4. Estimators

Let *x* and *n* be the total number of packets received by the current node and the total number of packets transmitted by the previous node, respectively, in a time frame for observation. That is, the number of packets lost in the time frame is n−x. We introduce three types of estimators for our numerical evaluations.

(1) Maximum likelihood estimator (MLE): The MLE, denoted by ${\hat{p}}_{\mathrm{MLE}}$, estimates *p* by maximizing the likelihood function. ${\hat{p}}_{\mathrm{MLE}}=\left(n-x\right)/n$ is a well-known result which can be obtained via derivative tests. This form collides with the sample average, so by the law of large numbers, ${\hat{p}}_{\mathrm{MLE}}\to p$ when n→∞ if *p* does not change over time.

(2) Minimax estimator: The minimax estimator achieves the smallest maximum risk among all estimators. With the popular mean squared error (MSE) as the risk function, it is a Bayes estimator with respect to the least favourable prior distribution. As studied in [90,91], such prior distribution is a beta distribution $\mathrm{Beta}(\sqrt{n}/2,\sqrt{n}/2)$. The minimax estimator of *p*, denoted by ${\hat{p}}_{\mathrm{MM}}$, is the posterior mean, which is $\frac{\sqrt{n}}{1+\sqrt{n}}\frac{n-x}{n}+\frac{1}{1+\sqrt{n}}\frac{1}{2}$, or equivalently, $\frac{n-x+0.5\sqrt{n}}{n+\sqrt{n}}$.

(3) Weighted Bayesian update: Suppose the prior distribution is Beta(a,b), where the hyperparameters can be interpreted as a pseudo-observation having *a* successes and *b* failures. Given a sample of *s* successes and *f* failures from a binomial distribution, the posterior distribution is Beta(a+s,b+f). To fade out the old samples captured by the hyperparameters, we introduce a scaling factor 0≤γ≤1 and let the posterior distribution be Beta(γa+s,γb+f). This factor can also prevent the hyperparameters from growing indefinitely. The estimation of *p*, denoted by ${\hat{p}}_{\mathrm{Bayes}}$, is the posterior mean with s=n−x and f=x, which is $\frac{\gamma a+n-x}{\gamma (a+b)+n}$. To prevent a bias when there are insufficient samples, we select a non-informative prior as the initial hyperparameters. Specifically, we use the Jeffreys prior, which is Beta(1/2,1/2).

**Figure 16:**
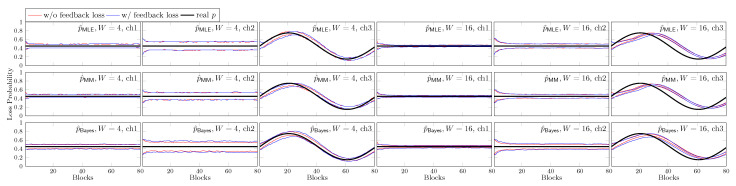
The 25 and 75% percentiles of the estimation of *p* by different schemes where |L|=M=4 in 1000 runs.

We first show the estimation of *p* by different schemes in Figure 16. We use BAR with (2) and |L|=M=4. The size of the time frame is *W* blocks. For ${\hat{p}}_{\mathrm{MLE}}$ and ${\hat{p}}_{\mathrm{MM}}$, the observations in the whole time frame have the same weight. For ${\hat{p}}_{\mathrm{Bayes}}$, the effect of each observation deceases exponentially faster. We consider an observation is out of the time frame when it is scaled into 10% of the original value. That is, we define the scaling factor by $\gamma =\sqrt[{W}]{0.1}$. In each subplot, the black curve is the real-time *p*. The red and blue curves are for the estimation without and with feedback loss, respectively. In each case, the two curves are the 25 and 75% percentiles from 1000 runs, respectively.

We can see that a larger *W* has a slower response to the change in *p* in ch3. Among the estimators, ${\hat{p}}_{\mathrm{Bayes}}$ has the fastest response speed as its observations in the time frame are not fairly weighted. Furthermore, although ch1 and ch2 have the same average loss rate, the estimation has a larger variance when the channel is bursty.

### 4.5. Throughput Evaluations

**Figure 17:**
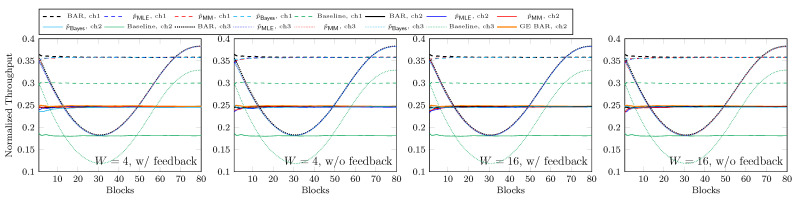
Throughput with estimated *p* via feedback where |L|=M=4.

As discussed in Section 4.2, the guessed *p* values have an insignificant impact on the throughput. We now show the throughput achieved by the estimation schemes in Figure 17. The parameters for the networks and BAR are the same as in Section 4.2. We do not wildly guess *p* here so it is no surprise that we can achieve nearly the same throughput as when we know the real *p* for ch1 and ch2. If we look closely, we can see from Figure 15 that for ch3, there is a small gap between the throughput of BAR when we know the real-time *p* and the one of BAR when using a constant *p*. Although the estimation may not be accurate at all times, we can now adapt to the change in *p* to finally achieve a throughput nearly the same as when we know the real-time *p*. On the other hand, whether the feedback is lost or not, the plots shown in Figure 17 are basically the same.

## 5. Conclusions

We proposed BAR in this paper which can adapt to variations in the incoming channel condition. In a practical perspective, we discussed how to calculate the components of BAR and how to solve BAR efficiently. We also investigated the impact of an inaccurate channel model on the throughput achieved by BAR. Our evaluations showed that

1. BAR is insensitive to the channel model: guessing the loss rate still outperforms BR.
2. For bursty channels, the throughput achieved by BAR with an independent loss model is nearly identical to one with the real channel model. That is, we can use the independent loss model for BAR in practice and apply the techniques in this paper to reduce the computational costs of BAR.
3. Feedback can slightly enhance the throughput for channels with a dynamic loss rate. This suggests that BAR works very well without the need of feedback. On the other hand, feedback loss barely affects the throughput of BAR. Therefore, we can send the feedback through a lossy channel without the need of retransmission. Unless we need to use an accurate estimated loss rate in other applications, we can use MLE with a small time frame for BAR to reduce the computational time.

These encouraging results suggest that BAR is suitable to be deployed in real-world applications.

One drawback of our proposed scheme is that we need to change the default behaviour of some intermediate network nodes, which can be a practical problem in existing networks. In fact, this is a common issue for all network coding schemes. Some routers have hard-wired circuits to efficiently handle heavy traffic, so it is unfeasible to deploy other schemes on them without replacing the hardware. For these heavy-loaded nodes, one may consider producing a hardware to speed up the network coding operations, e.g., [92,93], inducing extra costs on the deployment. On the other hand, the protocol for BNC is not standardized yet, meaning two parties may adopt BAR with incompatible protocols, thus restricting the application of BNC in public networks. However, it is not easy to build a consensus on the protocol, because there are still many research directions to improve the performance of BNC so the protocol design is subject to change in the near future.

## 6. Patents

The algorithms in Section 3 are variants of those that can be found in the U.S. patent 10,425,192 granted on 24 September 2019 [94]. The linear programming-based algorithm for BAR in Appendix H can be found in the U.S. patent 11,452,003 granted on 20 September 2022 [95].

## Figures and Tables

**Table 1:** Terminology used for batched network coding (BNC).

Terminology	Description
Batch	A small set of coded packets.
Batch size	The number of coded packets in a batch.
Rank of a batch	A measure of “useful” information (linearly independent packets) retained in the batch.
Expected rank of a batch	The expectation of the rank of the batch at the next network node.
Incoming rank distribution	The distribution of the ranks of the batches arriving at the current network node.
Throughput	The expectation of the rank distribution of the batches arriving at the destination node.
Recoding	The network coding operations restricted to the packets belonging to the same batch.
Recoded packets	The coded packets generated by recoding.
Recoder	The module that performs recoding.
Baseline recoding	A strategy that generates the same number of recoded packets per batch.
Adaptive recoding	A strategy that generates different number of recoded packets per batch.
Block	A set of batches.
Blockwise adaptive recoding	Applying adaptive recoding block by block.

**Table 2:** Frequently used notations in this paper.

Notation	Description
*M*	Batch size.
$r_{b}$	The rank of the batch *b*.
$t_{b}$	The number of recoded packets for batch *b*.
$E(r_{b},t_{b})$	The expected rank of batch *b* when its rank is $r_{b}$ at the current node and $t_{b}$ recoded packets are sent.
$\left(h_{0},\ldots ,h_{M}\right)$	The incoming rank distribution.
*p*	The packet loss rate in the independent packet loss model.
L	A block.
$t_{\max}^{L}$	The total number of recoded packets in block L.
$t_{\max}^{b}$	The maximum number of recoded packets allowed for batch *b*.
Binom(n,p)	The binomial distribution.
$B_{p}\left(t,i\right)$	The probability mass function of the binomial distribution Binom(t,1−p).
$\beta_{p}\left(t,r\right)$	The sum of the first *r* probability masses of Binom(t,1−p).
Beta(a,b)	The beta distribution.
$I_{x}\left(a,b\right)$	The regularized incomplete beta function.

## Data Availability

Not applicable.

## Abbreviations

The following abbreviations are used in this manuscript:

BAR	Blockwise adaptive recoding
IoT	Internet of Things
BNC	Batched network coding
RLNC	Random linear network coding
LDPC	Low-density parity-check code
BATS code	Batched sparse code
GE model	Gilbert–Elliott model
MLE	Maximum likelihood estimator
MSE	Mean squared error

## Appendix A. Accuracy of the Approximation $\zeta_{j}^{i,r}\approx \delta_{j,\min\{i,r\}}$

We demonstrate the accuracy of the approximation $\zeta_{j}^{i,r}\approx \delta_{j,\min\{i,r\}}$ by showing the percentage error of the expected rank function corrected to three decimal places when $q=2^{8}$, p=0.2 and $X_{t}∼\mathrm{Binom}\left(t,1-p\right)$ in Table A1. That is, the table shows the values

100∑i=0tti(1−p)ipt−i(min{r,i}−∑j=0min{i,r}jζji,r)∑i=0tti(1−p)ipt−i∑j=0min{i,r}jζji,r

for different *r* and *t*.

From the table, we can see that only three pairs of (r,t) have percentage errors larger than 0.1%, where they occur when r,t≤2. For all the other cases, the percentage errors are less than 0.1%. Therefore, such an approximation is accurate enough for practical applications.

**Table A1:** Percentage error when approximating expected rank functions.

*t*	r=1	r=2	r=3	r=4	r=5	r=6	r=7	r=8	r=9	r=10	r=11	r=12	r=13	r=14	r=15	r=16
1	0.39216	0.00153	0.00001	0.00000	0.00000	0.00000	0.00000	0.00000	0.00000	0.00000	0.00000	0.00000	0.00000	0.00000	0.00000	0.00000
2	0.13140	0.15741	0.00061	0.00000	0.00000	0.00000	0.00000	0.00000	0.00000	0.00000	0.00000	0.00000	0.00000	0.00000	0.00000	0.00000
3	0.03841	0.08032	0.08397	0.00033	0.00000	0.00000	0.00000	0.00000	0.00000	0.00000	0.00000	0.00000	0.00000	0.00000	0.00000	0.00000
4	0.01025	0.03091	0.05791	0.05042	0.00020	0.00000	0.00000	0.00000	0.00000	0.00000	0.00000	0.00000	0.00000	0.00000	0.00000	0.00000
5	0.00258	0.01024	0.02761	0.04398	0.03229	0.00013	0.00000	0.00000	0.00000	0.00000	0.00000	0.00000	0.00000	0.00000	0.00000	0.00000
6	0.00062	0.00308	0.01091	0.02502	0.03416	0.02155	0.00008	0.00000	0.00000	0.00000	0.00000	0.00000	0.00000	0.00000	0.00000	0.00000
7	0.00015	0.00087	0.00382	0.01147	0.02258	0.02685	0.01479	0.00006	0.00000	0.00000	0.00000	0.00000	0.00000	0.00000	0.00000	0.00000
8	0.00003	0.00023	0.00122	0.00457	0.01178	0.02023	0.02123	0.01036	0.00004	0.00000	0.00000	0.00000	0.00000	0.00000	0.00000	0.00000
9	0.00001	0.00006	0.00037	0.00165	0.00526	0.01182	0.01798	0.01686	0.00738	0.00003	0.00000	0.00000	0.00000	0.00000	0.00000	0.00000
10	0.00000	0.00002	0.00011	0.00055	0.00210	0.00585	0.01163	0.01585	0.01342	0.00532	0.00002	0.00000	0.00000	0.00000	0.00000	0.00000
11	0.00000	0.00000	0.00003	0.00017	0.00077	0.00257	0.00632	0.01125	0.01388	0.01070	0.00387	0.00002	0.00000	0.00000	0.00000	0.00000
12	0.00000	0.00000	0.00001	0.00005	0.00027	0.00103	0.00302	0.00665	0.01072	0.01208	0.00854	0.00284	0.00001	0.00000	0.00000	0.00000
13	0.00000	0.00000	0.00000	0.00002	0.00009	0.00038	0.00131	0.00344	0.00685	0.01009	0.01046	0.00682	0.00210	0.00001	0.00000	0.00000
14	0.00000	0.00000	0.00000	0.00000	0.00003	0.00013	0.00052	0.00160	0.00381	0.00693	0.00940	0.00901	0.00545	0.00156	0.00001	0.00000
15	0.00000	0.00000	0.00000	0.00000	0.00001	0.00004	0.00020	0.00069	0.00190	0.00412	0.00689	0.00866	0.00773	0.00436	0.00117	0.00000
16	0.00000	0.00000	0.00000	0.00000	0.00000	0.00001	0.00007	0.00028	0.00087	0.00219	0.00436	0.00677	0.00792	0.00660	0.00349	0.00088
17	0.00000	0.00000	0.00000	0.00000	0.00000	0.00000	0.00002	0.00010	0.00037	0.00106	0.00246	0.00454	0.00656	0.00718	0.00562	0.00279
18	0.00000	0.00000	0.00000	0.00000	0.00000	0.00000	0.00001	0.00004	0.00015	0.00048	0.00126	0.00271	0.00466	0.00629	0.00647	0.00476
19	0.00000	0.00000	0.00000	0.00000	0.00000	0.00000	0.00000	0.00001	0.00006	0.00020	0.00060	0.00147	0.00293	0.00471	0.00598	0.00579
20	0.00000	0.00000	0.00000	0.00000	0.00000	0.00000	0.00000	0.00000	0.00002	0.00008	0.00027	0.00073	0.00167	0.00311	0.00470	0.00563

## Appendix B. Proof of Lemma 1

We have the following properties when *a* and *b* are positive integers and 0≤x≤1 ([79], Equations 8.17.20 and 8.17.21):

(A1) Ix(a,b)−Ix(a+1,b)=a+b−1axa(1−x)b;

(A2) Ix(a,b+1)−Ix(a,b)=a+b−1bxa(1−x)b.

**Proof of Lemma 1(a)** It is trivial for i=0. For i>0, recall the recursive formula of binomial coefficients ([79], Equation 1.2.7):

t+1i=ti−1+ti,i=1,2,…,t.

Applying the formula, we have

  Bp(t+1,i)=t+1i(1−p)ipt+1−i=(1−p)ti−1(1−p)i−1pt−(i−1)+pti(1−p)ipt−i=(1−p)Bp(t,i−1)+pBp(t,i).

□

**Proof of Lemma 1(b).** Case I: t<r. By (10) and (12), $\beta_{p}\left(t+1,r\right)\leq 1=\beta_{p}\left(t,r\right)$, and the equality holds if and only if t+1≤r−1<r. Case II: t≥r>0. By (6) and (A1),

  βp(t,r)−βp(t+1,r)=Ip(t−r+1,r)−Ip(t−r+2,r)=tt−r+1pt−r+1(1−p)r>0.

Case III: t≥r=0. By (11), the equality always hold. □

**Proof of Lemma 1(c).** Case I: t<r. By (12), the equality always hold. Case II: t≥r>0. By (6) and (A2),

  βp(t+1,r+1)−βp(t,r)=Ip(t−r+1,r+1)−Ip(t−r+1,r)=trpt−r+1(1−p)r>0.

Case III: t≥r=0. By (10) and (11), $\beta_{p}\left(t,r\right)=0<\beta_{p}\left(t+1,r+1\right)$. □

**Proof of Lemma 1(d).** Case I: t=−1. By definition, $\beta_{p}\left(t,r+1\right)=\beta_{p}\left(t,r\right)=1$. Case II: t≥0. Recall that $\beta_{p}\left(t,r\right)$ is the partial sum of the probability mass of the binomial distribution Binom(t,1−p). By summing one more term, i.e., $\beta_{p}\left(t,r+1\right)$, the partial sum must be larger than or equal to $\beta_{p}\left(t,r\right)$. Note that $B_{p}\left(t,i\right)\neq 0$ when 0≤i≤t, so the equality holds if and only if $\beta_{p}\left(t,r\right)=1$ and if t<r by (12). □

**Proof of Lemma 1(e) and (f).** Inductively by Lemma 1(b), we have

(A3) $$\beta_{p}\left(t_{a}+u,r_{a}\right)\leq \beta_{p}\left(t_{a},r_{a}\right)\leq \beta_{p}\left(t_{a}-v,r_{a}\right)$$

for all a∈Λ where u,v are non-negative integers such that $t_{a}-v\geq -1$. By (10),

(A4) $$0\leq \min_{b\in \Lambda }\beta_{p}\left(t_{b},r_{b}\right)\leq \beta_{p}\left(t_{a},r_{a}\right)\leq \max_{b\in \Lambda }\beta_{p}\left(t_{b},r_{b}\right)\leq 1$$

for all a∈Λ. Combining (A3) and (A4), the proof is complete. □

## Appendix C. Proof of Lemma 3

By Lemma 3(a), we have $E\left(r,t+1\right)=E\left(r,t\right)+\left(1-p\right)\beta_{p}\left(t,r\right)$. If t<r, we have

$$\beta_{p}\left(t,r\right)=\sum_{i=0}^{r-1}B_{p}\left(t,i\right)=1,$$

which proves Lemma 3(a).

For Lemma 3(b), note that we have the initial condition

$$E\left(r,0\right)=B_{p}\left(0,0\right)\min\left\{r,0\right\}=0=\left(1-p\right)\sum_{j=0}^{(0)-1}\beta_{p}\left(j,r\right).$$

We can evaluate Lemma 2 recursively and obtain the first equality in Lemma 3(b).

By Lemma 3(a), we can show that when t<r, we have

(A5) E(r,t)=t(1−p).

This implies that when t≥r, we have

(A6) $$E\left(r,t\right)=\left(1-p\right)\left(r+\sum_{j=r}^{t-1}\beta_{p}\left(j,r\right)\right).$$

When t<r, the summation term $\sum_{j=r}^{t-1}\beta_{p}\left(j,r\right)$ in (A6) equals 0. So, we can combine (A5) and (A6) and give

$$E\left(r,t\right)=\left(1-p\right)\left(\min\left\{r,t\right\}+\sum_{j=r}^{t-1}\beta_{p}\left(j,r\right)\right).$$

## Appendix D. Proof of Theorem 2

Suppose $t_{m}>t_{n}$ for some $r_{m}<r_{n}$, i.e.,

(A7) $$t_{m}>t_{n}\geq r_{n}>r_{m}.$$

We define

$$t_{b}^{\prime }=\left\{\begin{array}{cc} t_{m} & \mathrm{if}\;b=n,\\ t_{n} & \mathrm{if}\;b=m,\\ t_{b} & \mathrm{otherwise} \end{array}\right.$$

for all b∈L. We consider the difference of

$$\begin{array}{cc} \;\;\sum_{b\in L}E\left(r_{b},t_{b}^{\prime }\right)-\sum_{b\in L}E\left(r_{b},t_{b}\right) & \\ =\left[E\left(r_{m},t_{n}\right)+E\left(r_{n},t_{m}\right)\right]-\left[E\left(r_{m},t_{m}\right)+E\left(r_{n},t_{n}\right)\right] & \\ =\left[E\left(r_{n},t_{m}\right)-E\left(r_{n},t_{n}\right)\right]+\left[E\left(r_{m},t_{n}\right)-E\left(r_{m},t_{m}\right)\right] & \\ =\left(1-p\right)\left[\left(\sum_{j=0}^{t_{m}-1}\beta_{p}\left(j,r_{n}\right)-\sum_{j=0}^{t_{n}-1}\beta_{p}\left(j,r_{n}\right)\right)+\left(\sum_{j=0}^{t_{n}-1}\beta_{p}\left(j,r_{m}\right)-\sum_{j=0}^{t_{m}-1}\beta_{p}\left(j,r_{m}\right)\right)\right] & \left(A8\right)\\ =\left(1-p\right)\left[\sum_{j=t_{n}}^{t_{m}-1}\beta_{p}\left(j,r_{n}\right)-\sum_{j=t_{n}}^{t_{m}-1}\beta_{p}\left(j,r_{m}\right)\right] & \\ =\left(1-p\right)\sum_{j=t_{n}}^{t_{m}-1}\left(\beta_{p}\left(j,r_{n}\right)-\beta_{p}\left(j,r_{m}\right)\right) & \\ >0, & \left(A9\right) \end{array}$$

where

- (A8) follows Lemma 3(b);
- (A9) follows Lemma 1(d) together with (A7).

The above result contradicts that ${\left\{t_{b}\right\}}_{b\in L}$ solves (8), which gives us that $t_{m}\leq t_{n}$ for all $r_{m}<r_{n}$.

Next, we suppose $t_{m}=t_{n}$ for some $r_{m}<r_{n}$, i.e.,

(A10) $$t_{m}=t_{n}\geq r_{n}>r_{m}.$$

We define

$$t_{b}^{\prime \prime }=\left\{\begin{array}{cc} t_{n}+1 & \mathrm{if}\;b=n,\\ t_{m}-1 & \mathrm{if}\;b=m,\\ t_{b} & \mathrm{otherwise} \end{array}\right.$$

for all b∈L. Moreover, we compare the difference of

$$\begin{array}{cc} \;\;\sum_{b\in L}E\left(r_{i},t_{i}^{\prime \prime }\right)-\sum_{b\in L}E\left(r_{i},t_{i}\right) & \\ =\left[E\left(r_{n},t_{n}+1\right)+E\left(r_{m},t_{m}-1\right)\right]-\left[E\left(r_{n},t_{n}\right)+E\left(r_{m},t_{m}\right)\right] & \\ =\left[E\left(r_{n},t_{n}+1\right)-E\left(r_{n},t_{n}\right)\right]-\left[E\left(r_{m},t_{m}\right)-E\left(r_{m},t_{m}-1\right)\right] & \\ =\left(1-p\right)[\beta_{p}\left(t_{n},r_{n}\right)-\beta_{p}\left(t_{m}-1,r_{m}\right)] & \left(A11\right)\\ \geq \left(1-p\right)[\beta_{p}\left(t_{m},r_{m}+1\right)-\beta_{p}\left(t_{m}-1,r_{m}\right)] & \left(A12\right)\\ >0, & \left(A13\right) \end{array}$$

where

- (A11) follows Lemma 3(a)
- (A12) follows (A10) and Lemma 1(d)
- (A13) follows Lemma 1(c) together with (A10).

This contradicts ${\left\{t_{b}\right\}}_{b\in L}$ and solves (8). Therefore, we have $t_{m}\neq t_{n}$ for all $r_{m}<r_{n}$.

Combining the two cases, the proof is complete.

## Appendix E. Performance Guarantee and Bounded Error of Algorithm 2

We start the discussion with the following theorem.

**Theorem** **A1.** *Let SOL and OPT be the solution given by Algorithm 2 and the optimal solution of* (8), *respectively, then*

$$\left\{\begin{array}{ccc} \mathrm{SOL} & \geq & (1-p)\mathrm{OPT},\\ \mathrm{OPT}-\mathrm{SOL} & \leq & \left(1-p\right)\sum_{b\in L}\sum_{j=r_{b}+\ell }^{r_{b}+\left|L\right|\ell^{\prime }-1}\beta_{p}\left(j,r_{b}\right), \end{array}\right.$$

*where $\ell^{\prime }=\left(t_{\max}^{L}-\sum_{b\in L}r_{b}\right)/\left|L\right|$ and $\ell =\lfloor \ell^{\prime }\rfloor$.*

**Proof.** We first show that the algorithm has a relative performance guarantee factor of 1−p. As stated in Theorem 3, when $t_{\max}^{L}\leq \sum_{b\in L}r_{b}$, the algorithm guarantees an optimal solution. Therefore, we only consider $t_{\max}^{L}>\sum_{b\in L}r_{b}$. Let ${\left\{t_{b}\right\}}_{b\in L}$ be the approximation given by the algorithm. Note that any linear combinations of *r*-independent vectors cannot obtain more than *r*-independent vectors. Therefore, the expected rank of a batch at the next hop must be no larger than the rank of the batch at the current hop, and, be non-negative. That is,

(A14) $$0\leq E\left(r_{b},t\right)\leq r_{b},\forall t\geq 0,b\in L.$$

This gives a bound of the optimal solution by

(A15) $$0\leq \mathrm{OPT}\leq \sum_{b\in L}r_{b}.$$

We consider the exact formula of the approximation:

$$\begin{array}{ccc} \;\;SOL & =\left(1-p\right)\sum_{b\in L}r_{b}+\left(1-p\right)\sum_{b\in L}\sum_{j=r_{b}}^{t_{b}-1}\beta_{p}\left(j,r_{b}\right) & \left(A16\right)\\ & \geq \left(1-p\right)\sum_{b\in L}r_{b} & \left(A17\right)\\ & \geq (1-p)\mathrm{OPT}, & \left(A18\right) \end{array}$$

where

- (A16) is stated in Lemma 3(b)
- (A17) holds as $\beta_{p}\left(j,r_{b}\right)\geq 0$ for all $j,r_{b}$, which is by (10);
- (A18) follows the inequality (A15).

Lastly, we show the bounded error. Let $\left\{t_{b}^{*}\right\}$ be a solution to (8). We write $t_{b}^{*}=r_{b}+\ell_{b}$ where $\ell_{b}\geq 0$ for all b∈L. Note that the constraint of (8), i.e., $\sum_{b\in L}t_{b}^{*}=t_{\max}^{L}$, suggests that

(A19) $$\ell_{b}\leq t_{\max}^{L}-\sum_{b\in L}r_{b}=\left|L\right|\ell^{\prime }.$$

On the other hand, it is easy to see that the approximation must either give $t_{b}=r_{b}+\ell$ or $t_{b}=r_{b}+\ell +1$. That is, we have $t_{b}\geq r_{b}+\ell$. By Lemma 3(b),we have

(A20) $$\mathrm{SOL}\geq \left(1-p\right)\sum_{b\in L}\left[r_{b}+\sum_{j=r_{b}}^{r_{b}+\ell -1}\beta_{p}\left(j,r_{b}\right)\right].$$

We consider the difference between OPT and SOL:

$$\begin{array}{cc} \;\;\mathrm{OPT}-\mathrm{SOL}\\ \leq \left(1-p\right)\sum_{b\in L}\left(\sum_{j=r_{b}}^{r_{b}+\ell_{b}-1}\beta_{p}\left(j,r_{b}\right)-\sum_{j=r_{b}}^{r_{b}+\ell -1}\beta_{p}\left(j,r_{b}\right)\right) & \left(A21\right)\\ =\left(1-p\right)\sum_{b\in L}\left(\sum_{\begin{array}{c} j=r_{b}+\ell ,\\ \ell_{b}>\ell \end{array}}^{r_{b}+\ell_{b}-1}\beta_{p}\left(j,r_{b}\right)-\sum_{\begin{array}{c} j=r_{b}+\ell_{b},\\ \ell_{b}<\ell \end{array}}^{r_{b}+\ell -1}\beta_{p}\left(j,r_{b}\right)\right)\\ \leq \left(1-p\right)\sum_{b\in L}\sum_{\begin{array}{c} j=r_{b}+\ell ,\\ \ell_{b}>\ell \end{array}}^{r_{b}+\ell_{b}-1}\beta_{p}\left(j,r_{b}\right) & \left(A22\right)\\ \leq \left(1-p\right)\sum_{b\in L}\sum_{j=r_{b}+\ell }^{r_{b}+\left|L\right|\ell^{\prime }-1}\beta_{p}\left(j,r_{b}\right), & \left(A23\right) \end{array}$$

where

- (A21) is the difference between the exact form of OPT by Lemma 3(b) after substituting the lower bound of SOL shown in (A20);
- the condition $\ell_{b}>\ell$ in the summation of (A22) can be removed, as we have $r_{b}+\ell_{b}-1<r_{b}+\ell$ if $\ell_{b}\leq \ell$;
- (A23) follows (A19) and the fact shown in (10) that the extra $\beta_{p}\left(j,r_{b}\right)$ terms are non-negative.

The proof is done. □

If the relative performance guarantee factor of 1−p is tight, we need both equalities in (A17) and (A18) to hold. First, by (10), we know that $\beta_{p}\left(j,r_{b}\right)$ is always non-negative. The equality in (A17) holds if and only if $\sum_{j=r_{b}}^{t_{b}-1}\beta_{p}\left(j,r_{b}\right)=0$ for all b∈L. The sum equals 0 only when

- $r_{b}=0$ and $t_{b}\geq 0$ according to (11); or
- $t_{b}-1<r_{b}$ which forms an empty sum.

The equality in (A18) holds if and only if $\mathrm{OPT}=\sum_{b\in L}E\left(r_{b},t_{b}^{*}\right)=\sum_{b\in L}r_{b}$. Note that (A14) shows that $E(r_{b},t_{b}^{*})$ is upper bounded by $r_{b}$. This implies that we need $E\left(r_{b},t_{b}^{*}\right)=r_{b}$ for all b∈L. When $t_{b}^{*}\leq r_{b}$, we can apply Lemma 3(a) to obtain $E\left(r_{b},t_{b}^{*}\right)=\left(1-p\right)t_{b}^{*}$, which equals $r_{b}$ if and only if $r_{b}=0$, as we assumed 0<p<1 in this paper. By Lemma 2, $E(r_{b},t)$ is a monotonic increasing function in terms of *t* for all $r_{b}\geq 0$. Therefore, when $r_{b}\neq 0$ we need $t_{b}^{*}>r_{b}$, which implies that $t_{\max}^{L}>\sum_{b\in L}r_{b}$. Then, the approximation will also give $t_{b}>r_{b}$ for some b∈L in this case, and the equality in (A17) does not hold.

That is, we have SOL=(1−p)OPT only when $r_{b}=0$ for all b∈L. In this case, we have SOL=OPT=0. In practice, the probability of having $r_{b}=0$ for all b∈L is very small. Therefore, we can consider that the bound is not tight in most cases but it guarantees that the approximation is good when the packet loss probability is small.

## Appendix F. Proof of Theorem 5

Let $B\left(a,b;y\right):=\int_{0}^{y}x^{a-1}{\left(1-x\right)}^{b-1}\;dx$ be the incomplete beta function. We have the beta function B(a,b):=B(a,b;1).

From (6), we have $\beta_{p}\left(t,r\right)=I_{p}\left(t-r+1,r\right)=\frac{B(t-r+1,r;p)}{B(t-r+1,r;1)}$. By direct calculation, the condition number is

(A24) |pdβp(t,r)dpβp(t,r)=pddp∫0pxt−r(1−x)r−1dxB(t−r+1,r;1)Ip(t−r+1,r)                 =pt−r+1(1−p)r−1B(t−r+1,r;1)Ip(t−r+1,r)                 =pt−r+1(1−p)r−1∫0pxt−r(1−x)r−1dx                 =pt−r+1∑j=0r−1(−1)jr−1jpj∫0pxt−r∑j=0r−1(−1)jr−1jxjdx                 =∑j=0r−1(−1)jr−1jpt−r+j+1∑j=0r−1(−1)jr−1j∫0pxt−r+jdx                 =∑j=0r−1(−1)jr−1jpt−r+j+1∑j=0r−1(−1)jr−1jpt−r+j+1/(t−r+j+1),

where the absolute value disappears as both numerator and denominator are non-negative. The first form of the condition number can be obtained by substituting $B\left(t-r+1,r;1\right)=\frac{(t-r)!(r-1)!}{t!}$ into (A24).

## Appendix G. Corrupted Heaps

In this appendix, we explain why we can omit two of the heap updates in Algorithm 3.

Before we start, we need some mathematical descriptions of the optimal solutions of BAR. Then, we will introduce our lazy evaluation technique on a modified heap that we called the corrupted heap.

To simplify the notations, we redefine $\beta_{p}\left(t_{b},r_{b}\right)$ as

βp(t,r)=0iftb≥tmaxb,1iftb≤min{rb,tmaxb}−1,∑i=0rb−1tbi(1−p)iptb−iotherwise.

in this appendix. In other words, $\beta_{p}\left(\cdot ,\cdot \right)$ is now a function of *b*. When $t_{b}\geq t_{\max}^{b}$, $\beta_{p}\left(t_{b},r_{b}\right)$ is the smallest possible value in the image of $\beta_{p}\left(\cdot ,\cdot \right)$. Therefore, the algorithms in this paper will not assign more recoded packets to the batch *b*.

### Appendix G.1. Optimality Properties of BAR

First, we introduce the following theorem that states a condition for non-optimality (or optimality after taking contraposition).

**Theorem** **A2.** *Let ${\left\{t_{b}\right\}}_{b\in L}$ be a feasible solution of* (9). *Then, ${\left\{t_{b}\right\}}_{b\in L}$ is not an optimal solution of* (9) *if and only if there exists two distinct batches κ and ρ with $t_{\rho }\geq 1$ such that $\left(1-p\right)\beta_{p}\left(t_{\kappa },r_{\kappa }\right)>\left(1-p\right)\beta_{p}\left(t_{\rho }-1,r_{\rho }\right)$.*

**Proof.** We first prove the sufficient condition. If ${\left\{t_{b}\right\}}_{b\in L}$ does not solve (9), then it means that there exists another configuration ${\left\{t_{b}^{\prime }\right\}}_{b\in L}$ which can give a higher objective value. Since $\sum_{b\in L}t_{b}=\sum_{b\in L}t_{b}^{\prime }=t_{\max}^{L}$, there exists distinct κ,ρ∈L such that $t_{\kappa }^{\prime }>t_{\kappa }$ and $t_{\rho }^{\prime }<t_{\rho }$. Note that $t_{\rho }^{\prime }\geq 0$ so we must have $t_{\rho }\geq 1$. We define

$$\Theta =\left\{\kappa :t_{\kappa }^{\prime }>t_{\kappa }\right\}\;\mathrm{and}\;\Phi =\left\{\rho :t_{\rho }^{\prime }<t_{\rho }\right\},$$

where

(A25) $$\sum_{\theta \in \Theta }\left(t_{\theta }^{\prime }-t_{\theta }\right)=\sum_{\phi \in \Phi }\left(t_{\phi }-t_{\phi }^{\prime }\right)>0.$$

Using the fact that ${\left\{t_{b}^{\prime }\right\}}_{b\in L}$ gives a larger objective value and by Lemma 3(b),we have

(A26) $$\sum_{\theta \in \Theta }\sum_{t=t_{\theta }}^{t_{\theta }^{\prime }-1}\left(1-p\right)\beta_{p}\left(t,r_{\theta }\right)>\sum_{\phi \in \Phi }\sum_{t=t_{\phi }^{\prime }}^{t_{\phi }-1}\left(1-p\right)\beta_{p}\left(t,r_{\phi }\right).$$

Now, we fix κ and ρ such that

$$\kappa \in \underset{\theta \in \Theta }{arg max}\beta_{p}\left(t_{\theta },r_{\theta }\right)\;\mathrm{and}\;\rho \in \underset{\phi \in \Phi }{arg min}\beta_{p}\left(t_{\phi }-1,r_{\phi }\right).$$

We have

$$\begin{array}{cc} \;\;\sum_{\theta \in \Theta }\left(t_{\theta }^{\prime }-t_{\theta }\right)\left(1-p\right)\beta_{p}\left(t_{\kappa },r_{\kappa }\right) & \\ \geq \sum_{\theta \in \Theta }\left(t_{\theta }^{\prime }-t_{\theta }\right)\left(1-p\right)\beta_{p}\left(t_{\theta },r_{\theta }\right) & \\ \geq \sum_{\theta \in \Theta }\sum_{t=t_{\theta }}^{t_{\theta }^{\prime }-1}\left(1-p\right)\beta_{p}\left(t,r_{\theta }\right) & \left(A27\right)\\ >\sum_{\phi \in \Phi }\sum_{t=t_{\phi }^{\prime }}^{t_{\phi }-1}\left(1-p\right)\beta_{p}\left(t,r_{\phi }\right) & \left(A28\right)\\ \geq \sum_{\phi \in \Phi }\left(t_{\phi }-t_{\phi }^{\prime }\right)\left(1-p\right)\beta_{p}\left(t_{\phi }-1,r_{\phi }\right) & \left(A29\right)\\ \geq \sum_{\phi \in \Phi }\left(t_{\phi }-t_{\phi }^{\prime }\right)\left(1-p\right)\beta_{p}\left(t_{\rho }-1,r_{\rho }\right), & \end{array}$$

where

- (A27) and (A29) follows Lemma 1(b);
- (A28) is the inequality shown in (A26).

Applying (A25), we have

$$\left(1-p\right)\beta_{p}\left(t_{\kappa },r_{\kappa }\right)>\left(1-p\right)\beta_{p}\left(t_{\rho }-1,r_{\rho }\right),$$

which proves the sufficient condition. Now we consider the necessary condition, where we have

(A30) $$\left(1-p\right)\beta_{p}\left(t_{\kappa },r_{\kappa }\right)>\left(1-p\right)\beta_{p}\left(t_{\rho }-1,r_{\rho }\right)$$

for some distinct κ and ρ. Let

$$t_{b}^{\prime }=\left\{\begin{array}{cc} t_{\kappa }+1 & \mathrm{if}\;b=\kappa ,\\ t_{\rho }-1 & \mathrm{if}\;b=\rho ,\\ t_{b} & \mathrm{otherwise} \end{array}\right.$$

for all b∈L. Then, we consider the following:

$$\begin{array}{cc} \;\;\sum_{b\in L}E\left(r_{b},t_{b}^{\prime }\right)\\ =\sum_{b\in L\setminus \{\kappa ,\rho \}}E\left(r_{b},t_{b}\right)+E\left(r_{\kappa },t_{\kappa }+1\right)+E\left(r_{\rho },t_{\rho }-1\right) & \\ =\sum_{b\in L\setminus \{\kappa ,\rho \}}E\left(r_{b},t_{b}\right)+E\left(r_{\rho },t_{\rho }-1\right)+\left[E\left(r_{\kappa },t_{\kappa }\right)+\left(1-p\right)\beta_{p}\left(t_{\kappa },r_{\kappa }\right)\right] & \left(A31\right)\\ >\sum_{b\in L\setminus \{\kappa ,\rho \}}E\left(r_{b},t_{b}\right)+E\left(r_{\kappa },t_{\kappa }\right)+\left[E\left(r_{\rho },t_{\rho }-1\right)+\left(1-p\right)\beta_{p}\left(t_{\rho }-1,r_{\rho }\right)\right] & \left(A32\right)\\ =\sum_{b\in L\setminus \{\rho \}}E\left(r_{b},t_{b}\right)+E\left(r_{\rho },t_{\rho }\right) & \left(A33\right)\\ =\sum_{b\in L}E\left(r_{b},t_{b}\right), & \end{array}$$

where

- (A31) and (A33) follow Lemma 3(a)
- (A32) follows (A30).

meaning that ${\left\{t_{b}\right\}}_{b\in L}$ is not an optimal solution of (9). □

Next, we define the sub-problems (A34) of (9) for $k\in \{0,1,\ldots ,t_{\max}^{L}\}$, which present the optimal substructure of (9).

(A34) $$\begin{array}{ccc} \max_{t_{b}\in \left\{0,1,2,\ldots \right\},\forall b\in L} & \; & \sum_{b\in L}E\left(r_{b},t_{b}\right)\\ s.t. & \; & \sum_{b\in L}t_{b}=k\\ \; & \; & t_{b}\leq t_{\max}^{b},\forall b\in L. \end{array}$$

Note that we assume $\sum_{b\in L}t_{\max}^{b}\geq t_{\max}^{L}\geq k$, or otherwise we should include more batches in the block.

We define a multiset $\Omega_{r}$ that collects the value of $\left(1-p\right)\beta_{p}\left(t,r\right)$ for all integers t≥0, i.e.,

$$\Omega_{r}=\left\{\left(1-p\right)\beta_{p}\left(t,r\right):t\in \left\{0,1,2,\ldots \right\}\right\}.$$

By Lemma 3(b),we have $E\left(r,t\right)=\left(1-p\right)\sum_{j=0}^{t-1}\beta_{p}\left(j,r\right)$. As E(r,t) is concave with respect to *t*, $\beta_{p}\left(t,r\right)$ is a monotonic decreasing function on *t* (also stated in Lemma 1(b)).

This implies the following lemma.

**Lemma** **A1.**
*E(r,t) equals the sum of the largest t elements in $\Omega_{r}$.*

**Proof.** By Lemma 3(b), $E\left(r,t\right)=\left(1-p\right)\sum_{j=0}^{t-1}\beta_{p}\left(j,r\right)$. By Lemma 1(b), $\beta_{p}\left(t,r\right)$ is a monotonic decreasing function on *t*. By (10) and (12), we have $\beta_{p}\left(0,r\right)=1\geq \beta_{p}\left(t,r\right)$ for all positive integers *t*. Therefore, E(r,t) is the sum of the largest *t* elements in $\Omega_{r}$. □

We fix a block L and define a multiset

$$\Omega :=\underset{b\in L}{⊎}\Omega_{r_{b}}=\left\{\left(1-p\right)\beta_{p}\left(t_{b},r_{b}\right):t_{b}\in \left\{0,1,2,\ldots ,t_{\max}^{b}-1\right\},b\in L\right\}.$$

If two batches a,b∈L have the same rank, i.e., $r_{a}=r_{b}$, then for all *t*, we have $\left(1-p\right)\beta_{p}\left(t,r_{a}\right)=\left(1-p\right)\beta_{p}\left(t,r_{b}\right)$. As Ω is a multiset, the duplicated values are not eliminated. Now, we have the following lemma to connect (A34) and Ω.

**Lemma** **A2.** *The optimal value of* (A34) *is the sum of the largest k elements in* Ω.

**Proof.** Let ${\left\{t_{b}\right\}}_{b\in L}$ solves (A34). We suppose the optimal value is not the sum of the largest *k* elements in Ω. However, Lemma A1 states that $E(r_{b},t_{b})$ equals the sum of the largest $t_{b}$ elements in $\Omega_{r_{b}}$ for all b∈L. This means that there exists two distinct batches κ,ρ∈L with $t_{\kappa }\leq t_{\max}^{b}-1$ and $t_{\rho }\geq 1$ such that $\left(1-p\right)\beta_{p}\left(t_{\kappa },r_{\kappa }\right)>\left(1-p\right)\beta_{p}\left(t_{\rho }-1,r_{\rho }\right)$. By setting $t_{\max}^{L}=k$, we can apply Theorem A2 which gives that ${\left\{t_{b}\right\}}_{b\in L}$ is not an optimal solution of (A34). The proof is completed by contradiction. □

We define a multiset $\Omega_{t_{\max}^{L}}^{\prime }$ which is a collection of the largest $t_{\max}^{L}$ elements in Ω. By Lemma A2, $\sum_{\omega \in \Omega_{t_{\max}^{L}}^{\prime }}\omega$ is the optimal value of (9). For any non-optimal solution ${\left(t_{b}^{\left(k\right)}\right)}_{b\in L}$, we define a multiset

$${℧}_{k}:=\left\{\left(1-p\right)\beta_{p}\left(t,r_{b}\right):t\in \left\{0,1,\ldots ,t_{b}^{\left(k\right)}-1\right\},b\in L\right\},$$

where the value $k\in \{0,1,\ldots ,t_{\max}^{L}-1\}$ is the number of elements in $\Omega_{t_{\max}^{L}}^{\prime }$ which are also contained in ${℧}_{k}$.

### Appendix G.2. Lazy Evaluations

We consider an iteration in the last **while** loop in Algorithm 3. Suppose we choose to increase $t_{b}$ by 1 and decrease $t_{a}$ by 1.

**Lemma** **A3.**
*If batch a is selected by the max-heap or batch b is selected by the min-heap in any future iteration, then the optimal solution is reached.*

**Proof.** Suppose batch *a* with key A is selected by the max-heap in a future iteration. Note that A was once the smallest element in ${℧}_{k}$ for some *k*. Therefore, at the current state where $k^{\prime }>k$, every element in ${℧}_{k^{\prime }}$ must be no smaller than A. Equivalently, we have $\left(1-p\right)\beta_{p}\left(t_{\kappa },r_{\kappa }\right)\leq \left(1-p\right)\beta_{p}\left(t_{\rho }-1,r_{\rho }\right)$ for all κ,ρ∈L. By Theorem A2, the optimal solution is reached. The min-heap counterpart can be proved in a similar fashion. □

Suppose we omit the update for the batch ρ in the heap. We call the key of the batch ρ a corrupted key, or the key of the batch ρ is corrupted. A key which is not corrupted is called an uncorrupted key. A heap with corrupted keys is called a corrupted heap. In other words, the key of a batch is corrupted in a corrupted max-heap if and only if the same batch was once the minimum of the corresponding original min-heap, and vice versa. As a remark, we do not have a guaranteed maximum portion of corrupted keys as an input. Furthermore, we do not adopt the carpooling technique. This suggests that the heap here is not a soft heap [97].

**Lemma** **A4.**
*If the root of a corrupted heap is a corrupted key, then the optimal solution is reached.*

**Proof.** We only consider a corrupted max-heap in the proof. We can use similar arguments to show that a corrupted min-heap also works. In a future iteration, suppose batch *a* is selected by the corrupted max-heap. We consider the real maximum in the original max-heap. There are three cases. Case I: batch *a* is also the root of the original max-heap. As the key of *a* is corrupted, it means that the batch was once selected by the corresponding min-heap. By Lemma A3, the optimal solution is reached. Case II: the root of the original max-heap is batch $a^{\prime }$ where the key of $a^{\prime }$ is also corrupted. Similar to Case I, batch $a^{\prime }$ was once selected by the corresponding min-heap, and we can apply Lemma A3 to finish this case. Case III: the root of the original max-heap is batch $a^{\prime \prime }$ where the key of $a^{\prime \prime }$ is not corrupted. In this case, the uncorrupted key of $a^{\prime \prime }$ is also in the corrupted max-heap. Note that the corrupted key of *a* is no larger than the actual key of *a* in the original max-heap. This means that the key of *a*, $a^{\prime \prime }$ and the corrupted key of *a* have the same value. It is equivalent to let the original max-heap select batch *a*, as every element in ${℧}_{k^{\prime }}$ must be no smaller than the key of $a^{\prime \prime }$, where $k^{\prime }$ represents the state of the current iteration. Then, the problem is reduced to Case I. Combining the three cases, the proof is completed. □

**Theorem** **A3.**
*The updates for batch a in the max-heap and batch b in the min-heap can be omitted.*

**Proof.** When we omit the updates, the heap itself becomes a corrupted heap. We have to make sure that when a batch with corrupted key is selected, the termination condition of the algorithm is also met. We can express the key of batch π in a corrupted max-heap and min-heap by $\beta_{p}\left(t_{\pi }+s_{\pi },r_{\pi }\right)$ and $\beta_{p}\left(t_{\pi }-1-u_{\pi },r_{\pi }\right)$, respectively, where $s_{\pi },u_{\pi }$ are non-negative integers. When $s_{\pi }$ or $u_{\pi }$ is 0, the key is uncorrupted in the corresponding corrupted heap. By Lemma 1(b), we have

$$\begin{array}{ccc} \beta_{p}\left(t_{\pi }+s_{\pi },r_{\pi }\right) & \leq & \beta_{p}\left(t_{\pi },r_{\pi }\right),\\ \beta_{p}\left(t_{\pi }-1,r_{\pi }\right) & \leq & \beta_{p}\left(t_{\pi }-1-u_{\pi },r_{\pi }\right). \end{array}$$

That is, the root of the corrupted max-heap is no larger than the root of the original max-heap. Similar for the min-heap. Mathematically, we have

(A35) $$\begin{array}{ccc} \max_{\pi \in L}\beta_{p}\left(t_{\pi }+s_{\pi },r_{\pi }\right) & \leq & \max_{\pi \in L}\beta_{p}\left(t_{\pi },r_{\pi }\right), \end{array}$$

(A36) $$\begin{array}{ccc} \min_{\pi \in L}\beta_{p}\left(t_{\pi }-1,r_{\pi }\right) & \leq & \min_{\pi \in L}\beta_{p}\left(t_{\pi }-1-u_{\pi },r_{\pi }\right). \end{array}$$

Suppose a corrupted key is selected. By Lemma A4, we know that the optimal solution is reached. Therefore, we can apply the contrapositive of Theorem A2 and know that

(A37) $$\left(1-p\right)\beta_{p}\left(t_{\kappa },r_{\kappa }\right)\leq \left(1-p\right)\beta_{p}\left(t_{\rho }-1,r_{\rho }\right)$$

for all κ,ρ∈L. We can omit the condition $t_{\rho }\geq 1$ because by (10) and (12), we have $\beta_{p}\left(-1,\cdot \right)=1\geq \beta_{p}\left(\cdot ,\cdot \right)$. The inequality (A37) is equivalent to

$$\max_{\pi \in L}\beta_{p}\left(t_{\pi },r_{\pi }\right)\leq \min_{\pi \in L}\beta_{p}\left(t_{\pi }-1,r_{\pi }\right).$$

We can mix this inequality with (A35) and (A36) to show that when a corrupted key is selected, we have

$$\max_{\pi \in L}\beta_{p}\left(t_{\pi }+s_{\pi },r_{\pi }\right)\leq \min_{\pi \in L}\beta_{p}\left(t_{\pi }-1-u_{\pi },r_{\pi }\right),$$

which is the termination condition shown in Algorithm 3 after we replaced the heaps into corrupted heaps. We just showed that once a corrupted key selected, the termination condition is reached. In the other words, before a corrupted key is selected, every previous selection must be an uncorrupted key. That is, the details inside the iterations are unaffected. If an uncorrupted key is selected where it also satisfies the termination condition, then no corrupted key is touched, and the corrupted heap still acts as a normal heap at this point. The correctness of the algorithm when using a corrupted heap is proven. Moreover, we do not need to mark which key is corrupted. This is, we can omitted the mentioned heap updates for a normal heap. □

We do not need to mark down which key is corrupted while the algorithm still works, so we can simply omit the mentioned updates as lazy evaluations. As there are two heaps in algorithm, we can reduce from four to two heap updates.

## Appendix H. Linear Programming Formulation of BAR

In [81], a distributionally robust optimization [98] for AR is formulated as a linear programming problem. It is based on an observation that when the expected rank function E(r,t) is concave with respect to *t*, we can reformulate it by

$$E\left(r,t\right)=\min_{i\in \{0,1,\ldots ,\bar{ı}\}}\left(\Delta_{r,i}t+\xi_{r,i}\right)$$

if we fix an artificial upper bound $t\leq \bar{ı}$, where $\Delta_{r,t}:=E\left(r,i+1\right)-E\left(r,i\right)$ and $\xi_{r,i}:=E\left(r,i\right)-i\Delta_{r,i}$. In (9), we implicitly have $t\leq t_{\max}^{L}$, so we can make use of this expression to write (9) as

$$\begin{array}{ccc} \max_{t_{b},e_{b}\geq 0,\forall b\in L} & \; & \sum_{b\in L}e_{b}\\ s.t. & \; & \sum_{b\in L}t_{b}=t_{\max}^{L}\\ \; & \; & t_{b}\leq t_{\max}^{b},\forall b\in L\\ \; & \; & e_{b}\leq E\left(r_{b},i\right)+\left(E\left(r_{b},i+1\right)-E\left(r_{b},i\right)\right)\left(t_{b}-i\right),\forall b\in L,\forall i\in \left\{0,1,\ldots ,t_{\max}^{L}\right\}, \end{array}$$

where $t_{b}$ is allowed to be a non-integer. A non-integer $t_{b}$ means that we first generate $\left\lfloor t_{b}\right\rfloor$ recoded packets, then we generate one more recoded packet with probability $t_{b}-\left\lfloor t_{b}\right\rfloor$. Note that there are $\left|L\right|t_{\max}^{L}$ constraints for $e_{b}$.

To turn such a non-deterministic solution into a deterministic one, we perform the following steps:

1. Collect the batches with non-integer recoded packets into a set *S*.
2. Calculate $R=\sum_{b\in S}\left(t_{b}-\left\lfloor t_{b}\right\rfloor \right)$. Note that *R* is an integer for BAR.
3. For every b∈S, remove the fractional part of $t_{b}$.
4. Randomly select *R* batches from *S* and add one recoded packet to each of these batches.

We have an integer *R* because $\sum_{b\in L}t_{b}=t_{\max}^{L}$. Furthermore, we have R<|S|. Referring to the idea of Algorithm 1, we have the same value of $\Delta_{r_{b},\left\lfloor t_{b}\right\rfloor }$ for all b∈S. After removing the fractional part of $t_{b}$ for all b∈S, it becomes the sub-problem (A34) (defined in Appendix G.1) with $k=t_{\max}^{L}-R$. The last step follows Algorithm 1 such that the output is a solution to (9) where $t_{b}$ for all b∈L are all integers.
